# Conservation and divergence of chemical defense system in the tunicate *Oikopleura dioica *revealed by genome wide response to two xenobiotics

**DOI:** 10.1186/1471-2164-13-55

**Published:** 2012-02-02

**Authors:** Fekadu Yadetie, Stephen Butcher, Hilde E Førde, Coen Campsteijn, Jean-Marie Bouquet, Odd A Karlsen, France Denoeud, Raghu Metpally, Eric M Thompson, J Robert Manak, Anders Goksøyr, Daniel Chourrout

**Affiliations:** 1 Sars International Centre for Marine Molecular Biology, University of Bergen, Bergen, Norway; 2 Department of Molecular Biology, University of Bergen, Bergen, Norway; 3 Department of Biology, University of Bergen, Bergen, Norway; 4 Department of Biology, University of Iowa, Iowa City, Iowa, USA; 5 Genoscope, Evry, France

## Abstract

**Background:**

Animals have developed extensive mechanisms of response to xenobiotic chemical attacks. Although recent genome surveys have suggested a broad conservation of the chemical defensome across metazoans, global gene expression responses to xenobiotics have not been well investigated in most invertebrates. Here, we performed genome survey for key defensome genes in *Oikopleura dioica *genome, and explored genome-wide gene expression using high density tiling arrays with over 2 million probes, in response to two model xenobiotic chemicals - the carcinogenic polycyclic aromatic hydrocarbon benzo[a]pyrene (BaP) the pharmaceutical compound Clofibrate (Clo).

**Results:**

*Oikopleura *genome surveys for key genes of the chemical defensome suggested a reduced repertoire. Not more than 23 cytochrome P450 (CYP) genes could be identified, and neither CYP1 family genes nor their transcriptional activator AhR was detected. These two genes were present in deuterostome ancestors. As in vertebrates, the genotoxic compound BaP induced xenobiotic biotransformation and oxidative stress responsive genes. Notable exceptions were genes of the aryl hydrocarbon receptor (AhR) signaling pathway. Clo also affected the expression of many biotransformation genes and markedly repressed genes involved in energy metabolism and muscle contraction pathways.

**Conclusions:**

*Oikopleura *has the smallest number of CYP genes among sequenced animal genomes and lacks the AhR signaling pathway. However it appears to have basic xenobiotic inducible biotransformation genes such as a conserved genotoxic stress response gene set. Our genome survey and expression study does not support a role of AhR signaling pathway in the chemical defense of metazoans prior to the emergence of vertebrates.

## Background

Animals protect themselves from xenobiotic or endogenous harmful chemicals by biotransformation and disposition of the compounds. Chemical defense mechanisms are well characterized in vertebrates. Recent comparisons of genomes from distantly related metazoans suggest conservation of the chemical defensome [1-3]. *Oikopleura dioica *belongs to larvacean tunicates that have considerable importance in the marine ecosystem and for the vertical flux of carbon in the form of discarded food-filtering house [4,5]. *Oikopleura *is surrounded by a complex mucopolysacharide house used to filter food particles from a large volume of water [6,7], and the therefore it can potentially be affected by marine pollution. *O. dioica *can be kept in culture for many generations and has a very short generation time (6 days at 15°C) [8]. Although tunicates are the closest living relatives of vertebrates [9], a recent study revealed profound divergence of genome architecture features in *Oikopleura *[10]. Its gene complement may have also markedly changed with multiple examples of ancestral genes failing detection, including genes involved in immunity [10]. In such a context of rapid genome evolution and peculiar life history, we decided to explore the genome for selected defensome genes and initiate investigations of *Oikopleura *responses to chemical stressors, for comparisons with invertebrate and vertebrate model systems.

The major components of chemical defensome of animals are phase I oxidative metabolic enzymes, phase II conjugating enzymes, phase III transporters, and transcription factors regulating the genes encoding biotransformation enzymes [2,11,12]. Xenobiotic inducible CYP families 1-4 and the transcription factors regulating them are among the most important and best studied defensome genes in vertebrates. Vertebrate CYP1 family enzymes play central roles in the metabolism of environmental toxicants such as polycyclic aromatic hydrocarbons (PAHs) [11,12]. Transcription factors of two classes, the aryl hydrocarbon receptor (AhR) and some nuclear receptor (NR) family members (NR1C and NR1I), regulate the expression of mammalian CYP families 1-4 [11,12]. AhR regulates CYP1 family genes (most importantly CYP1A1), the steroid and xenobiotic (or pregnane X) receptor (SXR/PXR) and constitutive androstane receptor (CAR) regulate CYP3A and CYP2B, and peroxisome proliferator-activated receptor alpha (PPARα) regulates CYP4A [11-14]. SXR and CAR are closely related nuclear receptors (NR1I) that are activated by a wide range of compounds including several clinically used drugs [15]. AhR is a major xenobiotic-sensing receptor that is activated by environmental pollutants such as dioxin and PAHs including benzo[a]pyrene (BaP) [16,17].

AhR and its dimerization partner AhR nuclear translocator (ARNT) are members of the family of basic-helix-loop-helix (bHLH)-Per-ARNT-Sim (PAS) domain transcription factors [16]. Upon ligand binding, AhR dimerizes with ARNT and activates the transcription of target genes that include CYP1 family genes (CYP1A1, CYP1A2, and CYP1B1 in mammals) and genes encoding phase II conjugating enzymes [16,18,19]. Hence the AhR signaling plays central roles in sensing and metabolic biotransformation of lipophilic toxicants in vertebrates [16,17].

AhR homologs are present in invertebrate genomes [20,21]. Invertebrate AhR plays developmental roles, and the xenobiotic receptor functions of AhR were suggested to have arisen in vertebrates [20,22]. AhR signaling pathway genes are interesting to examine in the tunicates since they were the last group diverging from vertebrates.

Other transcription factors involved in oxidative stress response include nuclear factor (erythroid-derived 2)-like 2 (Nrf2) and heat shock factors (HSFs). Oxidative biotransformation enzymes such as CYPs are often considered as double-edged swords because they may not only detoxify harmful chemicals but also create more toxic electrophilic metabolites that can damage cellular macromolecules such as DNA [12]. Generation of reactive oxygen species (ROS) intermediate metabolites leads to activation of Nrf2 which induces antioxidant enzyme genes including glutathione-s-transferases and glutamate-cysteine ligase subunits [23].

The first objective of our study was to search the *Oikopleura *genome for key candidate genes of chemical defense pathways; these include genes of relevant pathways, mainly CYPs, the transcription factors AhR, NR1I (SXR/PXR/CAR) and Nrf2 homologs. The second objective of the study was to examine the genome wide transcriptional responses to xenobiotic compounds using whole genome tiling arrays recently made available in *Oikopleura. Oikopleura *was exposed to two model xenobiotic compounds benzo[a]pyrene (BaP) and clofibrate (Clo) that are common environmental pollutants. BaP is a carcinogenic polycyclic aromatic hydrocarbon (PAH) that is a well characterized activator of AhR [24]. PAHs are ubiquitous environmental pollutants formed by incomplete combustion of organic matter [25]. Clo is a PPARα activator lipid-lowering pharmaceutical compound, which is also found as an environmental contaminant in surface waters [26].

## Results

### Small CYP complement and no detectable CYP1-like gene

**Figure 1:**
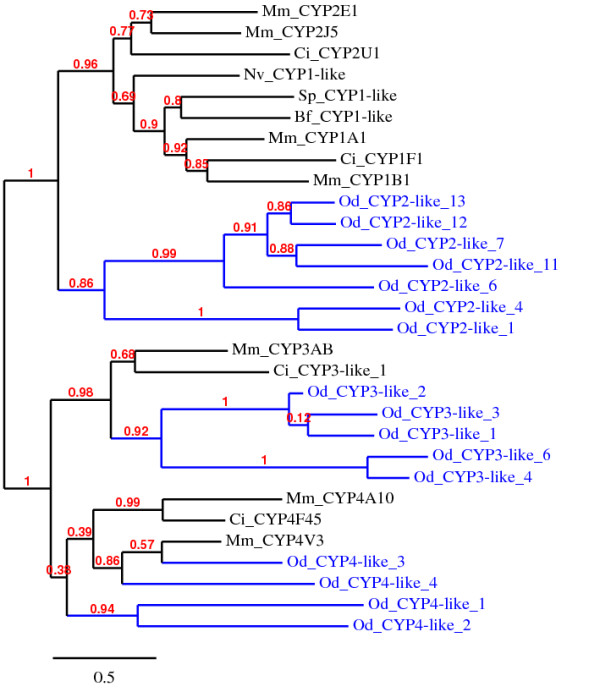
**Phylogenetic comparison of *Oikopleura *CYPs with representative CYPs from other organisms**. CYP1-like genes from Bf (*B. floridae*), Sp (*S. purpuratus*), Nv (*N. vectensis*) and CYP1-4 genes from Ci (*C. intestinalis*) and Mm (*M. musculus*) are included. Some *O. dioca *CYPs were removed to simplify the tree, but none of them cluster with CYP1sequences from other organisms. All the 23 *Oikopleura *CYPs and a larger set of species including some genes from [28] were also used to re-construct a larger phylogenetic tree with similar topology (Additional file 1, Figure S1). See Additional file 2 for the full list of *O. dioica *CYP proteins and their accessions. *O. dioica *(Od) sequences are shown in blue.

BLAST Searches using CYP1 family protein sequences from various vertebrates and the invertebrates did not result in CYP- like sequences in *O. dioica *geneome. We then searched the genome for all CYP sequences and unambiguously identified 23 unique genes (Additional file 1, Figure S1, Additional file 2). Thus, *Oikopleura *seems to have the smallest CYP gene complement of animal genomes sequenced thus far [27], http://drnelson.uthsc.edu/CytochromeP450.html. Phylogenetic analysis shows that none of the *Oikopleura *CYPs cluster with CYP1 family proteins from other organisms (Figure 1). A larger set of proteins and species including all the *23 Oikopleura *CYPs and many CYP1 protein sequences from various organisms [28] were also used to construct a phylogenetic tree of similar topology to the one shown in Figure 1, but none of the *Oikopleura *CYPs cluster with CYP1 sequences (Additional file 1, Figure S1). Thus, CYP1 genes are either lost or have diverged beyond recognition in *Oikopleura*. In contrast, CYP1-like genes are present in the genomes of *Ciona *and sea urchin [2,28]. *Oikopleura *has CYP3-like and CYP4-like genes but the 13 CYP2-like sequences do not cluster well with CYP2 family genes from other organisms (Additional file 1, Figure S1). The absence of CYP1-like genes appears consistent with the parallel absence of AhR signaling pathway (see below). Interestingly, our expression analysis showed that BaP and Clo induced CYP3-like-2 (CBY21750) and CYP2-like-11 (CBY24358) genes, respectively (Additional files 3 and 4).

### Lack of detection of AhR signaling pathway genes

**Figure 2:**
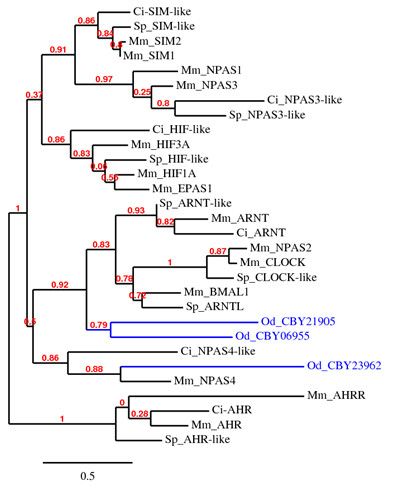
**Phylogenetic comparison of *Oikopleura *bHLH-PAS-domain proteins with representative proteins from other organisms**. The three *O. dioica *proteins (in blue) are indicated by accession numbers (see Additional file 2). See figure 1 legend for species abbreviations.

In mammals, bHLH-PAS domain proteins sense xenobiotics (AhR), oxygen (hypoxia-inducible factors/HIFs) and light (circadian locomoter output cycles protein kaput/clock) [18]. BLAST search in the *Oikopleura *genome and EST collections did not reveal a candidate ortholog for AhR. HMM searches for PAS domain proteins in predicted *Oikopleura *protein database and BLAST searches using various bHLH-PAS domain protein sequences of vertebrates and invertebrates identified three bHLH-PAS domain proteins in *Oikopleura*. One PAS domain protein identified (accession: CBY20245) is a potassium voltage-gated channel family like protein (not a member of bHLH-PAS domain proteins) and was not analyzed further. Phylogenetic comparison of the three *Oikopleura *bHLH-PAS domain proteins with representative homologs from mouse, *Ciona *and sea urchin showed none of them cluster with AhR (Figure 2). The failure to detect AhR and its repressor AhRR genes, as well as the downstream CYP1 family genes (Table 1) suggests the absence of an AhR mediated xenobiotic biotransformation signaling pathway in *Oikopleura*. In contrast, AhR orthologs are present in other invertebrates including the tunicate *C. intestinalis *[1,2,20,28].

**Table 1:** Comparison of numbers of selected defensome genes in genomes of *Oikopleura *and other organisms

Gene	*O. dioica*	*C. intestinalis*	*S. Purpuratus*	*M. musculus*
**AhR signaling**				
CYP1 genes	ND	5	10	3
AhR	ND	1	1 or 2	1
AhRR	ND	ND	ND	1
ARNT	1	1	1	1
**Nuclear receptors**				
NR1I (SXR/CAR/VDR)	6	2	ND	3
NR1H (LXR/FXR)	10	2	4	4
NR1C (PPAR)	ND	1	2	3
**Nrf2-signaling**				
Nrf2	1^a^	1	1	1
Keap1	1	1	1	1
MafK	1	1	1	1

^a ^clusters with both Nrf2 and Bach proteins (Additional file 1, Figure S2).

ND = Not detected. Data from [2,10,53,55] and NCBI database.

### *Oikopleura *NR1I-like and NR1H-like nuclear receptors

The absence of detected AhR sequence prompted us to inspect the *Oikopleura *genome for candidate xenobiotic sensing nuclear receptors. There are about 40 candidate nuclear receptors in *Oikopleura *(our unpublished data), suggesting significant lineage-specific expansion of the superfamily. SXR, CAR and VDR (vitamin D receptor) belong to NR1I subfamily of nuclear receptor genes. SXR and CAR serve as xenobiotic sensors that activate the transcription of some CYP genes in vertebrates [11,12]. In vertebrates, the NR1H subfamily of nuclear receptors liver-X-receptor (LXR) and farnesoid-X-receptor (FXR) are activated by sterols and bile acids, respectively [29]. LXR and FXR, also found in invertebrates [2], are potential targets of xenobiotic compounds. Genes encoding NR1I-like and NR1H-like nuclear receptors appear to be multiplied in the *Oikopleura *genome, even though the precise orthology relationships could not be clarified [10]. There are 6 NR1I and 10 NR1H (LXR/FXR-like) genes in the genome (Table 1) [10], and among these, there could be candidate xenobiotic receptors. No strong candidate for PPAR (NR1C) was found in *Oikopleura *(our unpublished data).

### Nrf2 mediated oxidative stress response pathway genes in *Oikopleura*

We further explored the *Oikopleura *genome for transcription factors involved in oxidative stress response, particularly the Nrf2-complex and found homologs of Nrf2, kelch-like ECH-associated protein 1 (Keap1), and small Maf proteins (Table 1, Additional file 1, Figure S2). Phylogenetic comparison of the *O. dioica *proteins with representative proteins from other organisms suggested a candidate Nrf2-homolog gene for *Oikopleura *(accession: CBY09280) although it clusters with both Nrf2 and Bach proteins (Additional file 1, Figure S2). Nrf2 is a member of cap 'n' collar-basic leucine-zipper (cnc-bZIP) proteins that heterodimerize with small Maf family proteins and activate transcription from anti-oxidant response elements (ARE) in promoters of anti-oxidant defense genes [30]. Nrf2 is normally bound to its suppressor Keap-1 and is inactive in the cytosol, but it dissociates under oxidative stress from Keap-1 and activates the transcription of target genes [23]. Mammalian Bach 1 and Bach 2 proteins are Nrf2-related factors that also heterodimerize with Maf proteins and mediate oxidative stress signaling [31]. Thus, although uncertainties remain due to the divergence of *Oikopleura *sequences, components of Nrf2 signaling appear to be present.

Other groups of transcription factors involved in the oxidative stress response include the heat shock factors (HSFs) [32]. In *Oikopleura*, there are two or three HSF5-like genes (not shown) and one gene very similar to both HSF1 and HSF4 (here referred to as HSF1/4) (CBY25079). The HSF1/4 and many HSP genes were strongly induced by BaP treatment (Additional file 1, Table S1, Additional file 3), and HSF binding sites could be identified in the promoter regions of many of the BaP induced HSP genes (not shown).

### Genes differentially regulated by BaP

BaP significantly changed the expression level of 762 annotated genes (336 genes up-regulated and 426 genes down-regulated) in *O. dioica *(Additional file 3). The list of affected genes includes several genes that are involved in drug metabolism and disposition. Xenobiotic Phase I and II biotransformation enzyme genes induced by BaP include genes encoding a cytochrome P450 family 3 (CYP3-like-2, CBY21750), carboxylesterase, glutathione S-transferase alpha (GSTα), thiosulfate sulfurtransferase and thioredoxin. Genes encoding Phase III transport proteins, ABC transporters and solute carrier proteins were also modulated. In BaP-treated animals, a co-regulation of genes in oxidative stress pathways was demonstrated by up-regulation of GSTα and the two subunits of glutamate-cysteine ligase (GCL), a rate limiting enzyme in glutathione synthesis (Additional file 1, Figure S3, Additional file 3). Another example of co- regulation is the simultaneous up-regulation of many heat-shock protein (HSP) genes and their transcription factor HSF1/4 gene [32,33] (Additional file 1, Table S1). HSP genes were among the most strongly induced genes (Additional file 1, Table S1, Additional file 3). Some putative HSP proteins of *Oikopleura *showed only low sequence similarity to mouse proteins (BLASTP E-value ≥ 0.001) and therefore were not included in the list of genes used for pathway analysis (see Methods).

### Pathways affected by BaP

In order to take advantage of the rich annotations of mammalian model organisms such as mouse, all predicted proteins of genes differentially regulated were annotated using BLAST searches against *M. musculus *proteome (BLASTP, e-value < 0.001). The best mouse hit for each *Oikopeleura *gene was then used in functional analyses using DAVID [34,35] and various tools in MetaCore (GeneGo) [36]. It is therefore important to interpret the pathway analyses data with caution since some pathways present in mammalian models may not be conserved or relevant in *Oikopleura*. For example, enriched disease pathways are not relevant in *Oikopleura*, although they may give insights into mechanisms of the underlying expression changes of the homolog genes in *Oikopleura*.

KEGG analyses using the mouse genes (best BLAST hits of *Oikopleura *genes differentially regulated by BaP) revealed significantly enriched top KEGG pathways such as *focal adhesion, ECM-receptor *interaction, *pathways in cancer, small cell lung cancer *and *nitrogen metabolism *(Table 2). Pathways related to *cancer *reflect the mouse annotations, but some of the genes in the pathways appear to be involved in conserved cellular processes such as DNA damage and oxidative stress responses.

**Table 2:** Significantly enriched KEGG pathways after BaP exposure

KEGG pathway term	Count	Pvalue	Benjamini	FDR
Focal adhesion	27	1.65E-08	2.57E-06	0.00002
ECM-receptor interaction	14	1.09E-05	8.48E-04	0.01308
Pathways in cancer	27	1.69E-04	8.76E-03	0.20313
Small cell lung cancer	12	3.06E-04	1.19E-02	0.36717
Nitrogen metabolism	6	1.39E-03	4.26E-02	1.66250

**Figure 3:**
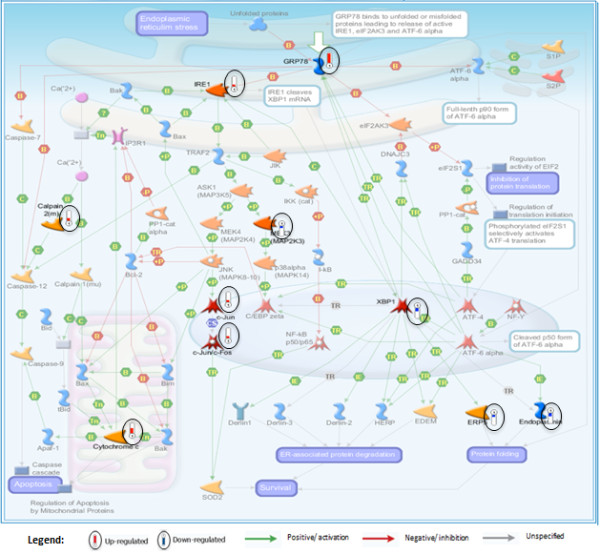
**The top scored pathway map affected by BaP "Apoptosis and survival_Endoplasmic reticulum stress response"**. Mouse homologs of genes differentially regulated by BaP in *Oikopleura *are indicated on the pathway map by thermometer like symbols (encircled for clarity). See Additional file 5 for detailed figure legend.

**Figure 4:**
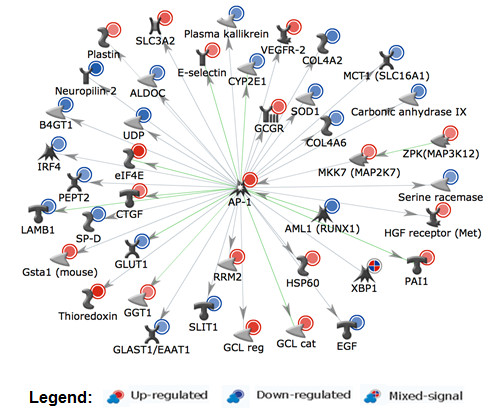
**AP-1 centered network of BaP-regulated genes sets**. MetaCore *Transcription Regulation *algorithm with default settings was used for generation of biological networks. For clarity, only genes with direct connections with AP-1 and marked by the GO Process terms "Response to Stress" and "Response to Chemical Stimulus" are shown. The network objects were rendered grey for better visualization of expression data. See Additional file 5 for detailed figure legend.

MetaCore enrichment analyses showed most enriched BaP affected pathways are involved in apoptosis, oxidative stress response, immune response, protein folding and cell adhesion (Table 3A and 3B). The top scored pathway map (*Apoptosis and survival_Endoplasmic reticulum stress response*) is shown in Figure 3 with the BaP affected genes indicated. In MetaCore *Interactome analysis *option, networks can be built to see interconnectedness within a data set. Genes in the input list can have physical and functional interactions (such as binding, catalytic, phosphorylation, transcriptional regulation) that are used by the algorithm to build sub-networks ranked by relative enrichment with the uploaded genes and relative saturation of networks with canonical pathways [36]. A detailed legend for MetaCore pathway maps and network objects is shown in Additional file 5. The network building tool in MetaCore was used to create networks using the *Transcription Regulation *algorithm, and resulted in a list of ranked transcription factor centered networks (Additional file 6). One of the BaP-induced key transcription factors, JunD is a functional component of the AP-1 transcription factor complex. AP-1 is one of the top ranked transcription factors (Additional file 6) and many oxidative stress response genes including GCLm, GCLc and thioredoxin that are up-regulated by BaP are in the AP-1 network (Figure 4).

**Table 3:** Enriched Pathway Maps (A) and Process Networks (B) for genes differentially regulated by BaP

Rank	A. Pathway Maps	pValue
1	Apoptosis and survival_ER stress response pathway	6.5E-06
2	NRF2 regulation of oxidative stress response	7.7E-06
3	Role of alpha-6/beta-4 integrins in carcinoma progression	1.5E-05
4	Mechanisms of CFTR activation by S-nitrosoglutathione	1.8E-05
5	Immune response_IL-12 signaling pathway	1.8E-05
6	Oxidative stress_Role of ASK1 under oxidative stress	1.9E-05
7	Development_TGF-beta-dependent induction of EMT via MAPK	2.1E-05
8	Development_Glucocorticoid receptor signaling	2.4E-05
9	Cytoskeleton remodeling_TGF, WNT and cytoskeletal remodeling	2.4E-05
10	Immune response_IL-7 signaling in T lymphocytes	4.1E-05
	**B. Networks**	
1	Protein folding_Response to unfolded proteins	2.9E-08
2	Cell adhesion_Platelet-endothelium-leucocyte interactions	9.0E-06
3	Protein folding_ER and cytoplasm	3.8E-05
4	Development_Blood vessel morphogenesis	4.7E-05
5	Reproduction_Spermatogenesis, motility and copulation	5.1E-05
6	Cell adhesion_Cell-matrix interactions	5.2E-05
7	Signal transduction_ESR1-nuclear pathway	6.3E-05
8	Development_Regulation of angiogenesis	2.8E-04
9	Proteolysis_Connective tissue degradation	5.3E-04
10	Signal transduction_NOTCH signaling	6.0E-04

To explore interactions within the set of BaP modulated genes, networks were generated in MetaCore using *Analyze *algorithm with default settings. In the top ranking networks appear genes that are also part of the enriched pathways and processes (Table 3A and 3B, Additional file 7). For example many stress response genes including those involved in the GO Biological Process "endoplasmic reticulum unfolded protein response" are represented in the third ranking network (Additional file 1, Figure S4).

### Comparison of BaP induced responses between *Oikopleura *and human cells

In order to assess conservation of responses to BaP in mammals and *Oikopleura*, we compared pathways significantly affected by BaP in this experiment and in a gene set from a microarray experiment using human macrophages [37]. Many oxidative stress and apoptosis related pathways were affected in both *Oikopleura *and human cells (Additional file 1, Figure S5 A and B) suggesting conservation of responses to genotoxic carcinogenic compounds. However, consistent with our genome survey results that showed absence of AhR signaling genes (Figure 2), the *AhR signaling pathway *was not enriched in *Oikopleura*, but among the top enriched pathways in human cells (Additional file 1, Figure S5B). Other major differences in the top 10 pathways enriched are related to cell-cycle regulation, which were among the top over-represented in human cells but not in *Oikopleura *(Additional file 1, Figure S5B). This could be because of species specific differences in cell-cycle control mechanisms, which in *Oikopleura *involves endocycling, and not necessarily mitosis [38]. Generation of transcription factors centred over-connected hubs in MetaCore (*Transcription Regulation *algorithm) resulted in a largely similar list of transcription factors for *Oikopleura *and human BaP-regulated gene sets (Additional file 6, not shown). Not surprisingly, AhR was in the human list of transcription factor networks (not shown), but not in the *Oikopleura *list, suggesting lack of modulated AhR target genes in the latter. Another notable difference is that BaP appears to provoke stronger endoplasmic reticulum (ER) stress response in *Oikopleura *as suggested by significant over-representation of *Apoptosis and survival_Endoplasmic reticulum stress response *pathway in *Oikopleura *but not human cells (Additional file 1, Figure S5B). It is not clear why *Endoplasmic reticulum stress response pathway *is responding more in *Oikopleura*. This pathway can be adaptive, promoting survival or it can lead to induction of apoptosis [39]. However, *Apoptosis *genes respond equally in both *Oikopleura *and the human macrophage cells (Additional file 1, Figure S5A). ER stress response can also explain the significant enrichment of *Cystic fibrosis disease *as one of the processes affected by BaP in *Oikopleura *but not human cells (Additional file 1, Figure S5A). Some pathways and processes such as human diseases are enriched here because the pathway analyses were done on mouse homologs of differentially regulated *Oikopleura *genes, but the underlying changes in gene expression or cellular processes can be relevant also in *Oikopleura*. For example, inspection of the modulated genes that led to the enrichment of C*ystic fibrosis disease *reveals that they are involved in ER stress response (e.g. HSPs). ER stress response is thought to contribute to mechanisms leading to the development of lung fibrosis in mice [40].

### Genes and pathways affected by Clo

Treatment of *O. dioica *with Clo resulted in differential regulation of 630 genes, with 166 genes up-regulated and 464 genes down-regulated (Additional file 4). Genes involved in the xenobiotic defense system in vertebrates were part of the list, including biotransformation enzyme genes such as CYP2-like-11 gene (CBY24358), conjugating enzyme genes such as GSTα, and some ABC transporters and solute carrier proteins (Additional file 4). Superoxide dismutase and many ABC transporters were also down-regulated by Clo.

**Figure 5:**
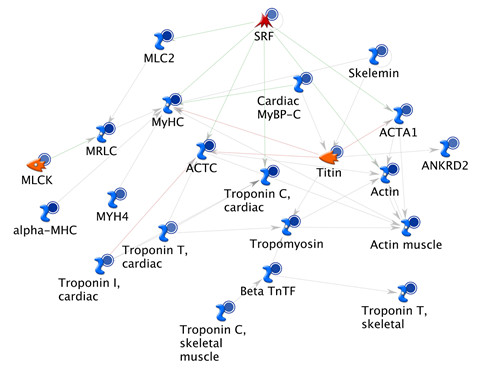
**The top scored process network "Muscle contraction" from Clo-modulated gene list**. Mouse homologs of Clo-regulated genes were used for enrichment analysis in MetaCore. All genes shown in the network are down regulated (indicated with blue circles). For clarity, genes that are not in Clo-modulated gene list were omitted. See Additional file 5 for detailed figure legend.

The list of genes differentially regulated by Clo was used in various functional analyses using KEGG and MetaCore (GeneGo) as described above for BaP gene set. Clo affected mainly muscle contraction and energy metabolism pathways (Tables 4 and 5, Figure 5). Among the significantly enriched pathways, *ECM-receptor interaction *and *Focal adhesion *are also affected by BaP (Table 2). Clo had orchestrated effects on main energy pathways such as *Glycolysis/Gluconeogenesis, Oxidative Phosphorylation *and *Citrate cycle *(*TCA cycle*) (Table 4). While most pathways affected by Clo are presumably conserved between *Oikopleura *and mouse, some such as the disease pathways (Table 4) may be less relevant for *Oikopleura*. Remarkably, nearly all the genes affected by Clo in the enriched muscle component and energy pathways were down-regulated (Figure 5, not shown), suggesting functional suppression of energy metabolism and motility. This appears consistent with the reduced motility of the animals observed for the highest concentration of Clo (not shown).

**Table 4:** Significantly enriched KEGG pathways after Clo exposure

Term	Count	PValue	Benjamini	FDR
ECM-receptor interaction	12	8.28E-05	1.15E-02	0.098
Valine, leucine and isoleucine degradation	9	1.15E-04	7.99E-03	0.135
Cardiac muscle contraction	11	2.31E-04	1.07E-02	0.273
Arginine and proline metabolism	9	3.19E-04	1.11E-02	0.376
Glycolysis/Gluconeogenesis	10	3.74E-04	1.04E-02	0.441
Hypertrophic cardiomyopathy (HCM)	11	4.27E-04	9.92E-03	0.503
Focal adhesion	17	8.40E-04	1.67E-02	0.987
Dilated cardiomyopathy	11	8.86E-04	1.54E-02	1.040
Alzheimer's disease	16	9.98E-04	1.54E-02	1.171
Huntington's disease	16	1.06E-03	1.47E-02	1.238
Oxidative phosphorylation	13	1.21E-03	1.53E-02	1.418
Parkinson's disease	13	1.48E-03	1.71E-02	1.727
Propanoate metabolism	6	2.80E-03	2.98E-02	3.260
Citrate cycle (TCA cycle)	6	3.26E-03	3.21E-02	3.774

**Table 5:** Enriched Map Folders (A) and Process Networks (B) for genes differentially regulated Clo

Rank	A. Map Folders	pValue
1	Tissue remodeling and wound repair	1.7e-6
2	Energy metabolism and its regulation	1.7e-5
3	Inflammatory response	1.2e-4
4	Cell differentiation	2.6e-4
5	Protein degradation	3.8e-4
6	Calcium signaling	1.0e-3
7	Vasodilation	1.8e-3
8	Lipid Biosynthesis and regulation	4.2e-3
9	Vasoconstriction	4.7e-3
10	Blood clotting	9.8e-3
	**B. Networks**	
1	Muscle contraction	9.4E-15
2	Cytoskeleton_Actin filaments	8.0E-10
3	Development_Skeletal muscle development	2.2E-09
4	Cytoskeleton_Regulation of cytoskeleton rearrangement	1.9E-07
5	Development_Cartilage development	6.5E-05
6	Cytoskeleton_Intermediate filaments	7.9E-05
7	Cell adhesion_Integrin-mediated cell-matrix adhesion	3.2E-04
8	Cytoskeleton_Cytoplasmic microtubules	4.8E-04
9	Cardiac development_BMP_TGF_beta_signaling	5.6E-04
10	Cell adhesion_Cell-matrix interactions	8.4E-04

To see interaction among genes differentially regulated by Clo, the network building tool in Metacore was used (with *Analyze *algorithm). This resulted in a list of ranked interaction networks (Additional file 8). Consistent with the functional ontology top list (Tables 4 and 5), the first five networks were enriched in genes involved in oxidative phosphorylation and muscle contraction (Additional file 1, Figure S6 A and B).

### Comparison of BaP and Clo affected genes and pathways

The major pathways and processes specifically affected by BaP are related to its known genotoxic oxidative stress effects such as *apoptosis, DNA-damage response, mitogenic signaling *and *response to un-folded proteins *(Additional file 1, Figure S5 A and B). Pathways and processes specifically affected by Clo are *energy metabolism *and *its regulation, muscle contraction, actin filaments, skeletal muscle development *and *regulation of cytoskeleton rearrangement *(Additional file 1, Figure S7 A and B). Enriched pathways and processes significantly affected by both BaP and Clo are *tissue remodelling and wound repair, inflammatory response, cell differentiation *and *cell adhesion *(Additional file 1, Figure S7 A and B).

Comparison of GO Cellular Component (CC) in MetaCore generated different top lists of localizations for BaP and Clo-regulated gene products, reflecting differences in enriched pathways (Additional file 1, Figure S7C). The only localization that is significantly enriched by BaP alone is *Golgi apparatus *(Additional file 1, Figure S7C). Several localization GO terms were significantly enriched by Clo but not BaP. These include muscle components (e.g. *contractile fibre *and *myofibril*) and organelles parts (e.g. *mitochondrial matrix*), consistent with top enriched muscle contraction and energy metabolism pathways, respectively (Tables 4 and 5, Figure 5, Additional file 1, Figure S7 A-C). The two compounds also share top list of GO localizations, mainly extracellular region (Additional file 1, Figure S7C), because both affected many extracellular proteins, possibly including yet un-characterized structural proteins of the house which is composed of more than 20 glycoproteins proteins [6,7]. Although not included in pathway and GO analyses, the extracellular house component proteins (oikosins) of *Oikopleura *are among the genes down-regulated by both compounds (Additional file 1, Table S2). GO analysis also allocated 35% of 125 genes down-regulated by both compounds (Additional file 1, Table S3A) to the GO-CC term *extracellular region *and 59% of them are annotated as *glycoproteins*. Only 24 genes were up-regulated by both chemicals (Additional file 1, Table S3B). Thirteen genes were oppositely regulated by BaP and Clo (Additional file 1, Table S3C).

### Validation of tiling array results by qPCR

**Figure 6:**
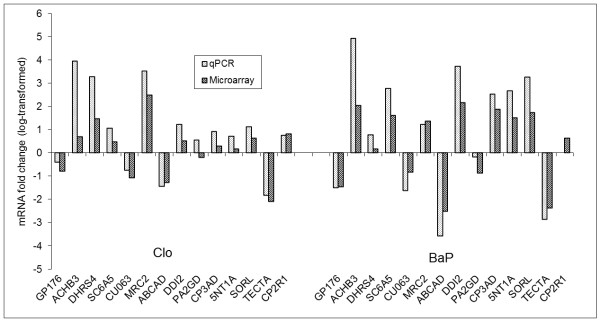
**Comparison of expression levels measured with tiling array hybridization and qPCR**. Log2 transformed fold changes in mRNA levels (treated/control) were plotted for both array and qPCR. Fold changes in mRNA levels measured by microarray and qPCR showed good correlation (Pearson's correlation coefficient, r = 0.93). Abbreviations of gene names are given in Additional file 1, Table S4.

Fourteen genes differentially regulated in tiling arrays were selected for validation using qPCR (Additional file 1, Table S4). The results showed good agreement between the two expression assays (Pearson's correlation coefficient, r = 0.93) (Figure 6). The directions of changes in log transformed fold-changes in expression (treated/control) using the two methods were consistent for all genes with the exception of PA2GD in Clo-treated samples and CYP2R1 in BaP-treated samples (Figure 6). Good correlation between the two methods was achieved because in the tiling array design, each gene in the genome was represented by several overlapping probes along its entire length (see Additional file 1, Figure S3) resulting in a robust statistical quantification of expression levels as described in methods section.

## Discussion

We searched the *Oikopleura *genome for homologs of key genes involved in xenobiotic response pathways. Using whole genome tiling arrays, we have recorded transcriptional responses of *Oikopleura *to two model xenobiotic compounds (BaP and Clo). *Oikopleura *appears to have the smallest CYP complement of sequenced animal genomes and has no detectable CYP1 family genes. AhR, which is the transcriptional regulator of CYP1 family genes in vertebrates, is also undetectable in *Oikopleura*. Thus, the AhR signaling pathway may not exist in *Oikopleura*. It is important to stress that although AhR is present in other invertebrates including *Ciona *and sea urchin [2,28], its involvement in xenobiotic defense has not been shown. AhR homologs in invertebrates examined thus far do not bind the prototypical ligand dioxin [20,22]. However, AhR has also developmental functions that are considered to be more ancestral [20,22]. Hence, the xenobiotic receptor function of AhR could be a vertebrate innovation as suggested before [20,22]. The absence of AhR signaling in *Oikopleura *re-enforces this conclusion and further suggests it might be disposable also in developmental mechanisms, at least in *Oikopleura*.

**Figure 7:**
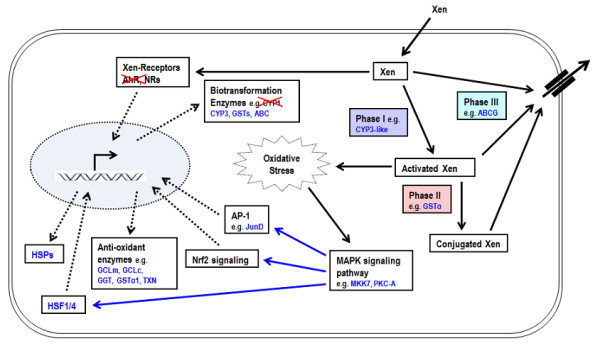
**Schematic summary of major elements of cellular defense system that may operate against xenobiotic compounds in *Oikopleura***. A lipophilic xenobiotic (Xen) compound such as BaP entering the cell may activate xenobiotic receptors AhR or NRs. The activated receptors induce transcription of phase I, phase II and phase III biotransformation genes that metabolize the compound. Phase I biotransformation may create ROS, inducing oxidative stress that can possibly activate other transcription factors (AP-1, Nrf2 and HSF1/4) via MAPK signaling resulting in induction of genes for anti-oxidant enzymes and HSPs. Phase III efflux transporters may pump out the compound and its biotransformation product. Examples of genes that are up-regulated by BaP in *Oikopleura *are shown in blue color. AhR and CYP1 are crossed out in red to show the absence of the genes in *Okopleura *genome. Solid black arrows indicate biotransformation such as binding, activation and transport. Dotted black arrows indicate increase in transcript or protein synthesis. Solid blue arrows indicate activation of transcription factors through MAPK signaling. Figure adapted and modified from [2].

Both BaP and Clo exposures affected the expression level of genes that participate in xenobiotic biotransformation and stress responses in vertebrates. BaP markedly changed the expression of many oxidative stress responsive genes. Phase I oxidative biotransformation reactions generate reactive electrophilic metabolites of BaP that result in oxidative stress in vertebrates [41]. The up-regulation of many biotransformation and anti-oxidant enzyme genes reveals similarities in oxidative stress responses in *Oikopleura *and vertebrates (Figure 7) [37,42,43]. Furthermore, analysis using BaP-modulated genes in *Oikopleura *and human macrophage cells [37] showed an enrichment of shared pathways such as DNA-damage response and apoptosis (Additional file 1, Figure S5 A and B), suggesting a conservation of response to genotoxic stress. A notable exception is the AhR signaling pathway, which was activated by BaP treatment in human cells but not in *Oikopleura *(Additional file 1, Figure S5B).

The similarities in BaP-modulated genes and pathways in both vertebrates and *Oikopleura *are surprising in the absence of AhR and CYP1 family genes in the latter. BaP is converted to more toxic intermediate metabolites by CYP1 enzymes in vertebrates and thus, CYP1 inducing AhR is required for carcinogenicity of BaP [17,24]. Although CYP1-like genes could not be detected in *Oikopleura*, it is possible that other CYP genes such as the CYP3-like (CYP3-like-2, CBY21750) enzyme induced here are involved in generation of reactive metabolites of BaP.

In contrast to BaP, responses to Clo treatment do not seem consistent with its effects in mammals, possibly because PPARs or NR1C-like genes are absent in the *Oikopleura *genome. In vertebrates, PPARα ligands such as Clo induce many fatty acid beta oxidation and oxidative phosphorylation enzymes [44,45]. In contrast to vertebrates, Clo down-regulated several genes in energy metabolism pathways in *Oikopleura*. Our results in *Oikopleura *thus far do not support conserved PPARα-dependent pathways but suggest general stress effects of Clo.

Some genes and pathways affected in this study appear to be compound specific and others are likely to be general toxic stress responses. For example some top pathways affected by BaP are shared by both *Oikoplera *and the human macrophage cells (Additional file 1, Figure S5 A and B) suggesting that they are BaP specific responses. Different CYP genes induced by the two compounds (a CYP3-like gene by BaP and a CYP2-like gene by Clo), are examples of compound specific biotransformation enzymes. Pathways affected by both compounds in *Oikopleura *such as *cell adhesion, tissue remodelling and wound repair *(Additional file 1, Figure S7 A and B) appear to be general stress responses. Both BaP and Clo also resulted in down-regulation of oikosins that are components of the house [6,7]. *Oikopleura *invests a significant portion of energy in the synthesis of its food-filtering house, composed of more than 20 proteins [6,7,46]. It is possible that stress and reduced food intake (a likely consequence of reduced house production) triggers energy saving adaptive responses such as reduced muscle contraction and lower energy metabolism in Clo-treated animals. These findings suggest that BaP and Clo pollution could be detrimental to growth and survival of *Oikopleura *in its environment.

The mechanisms of transcriptional regulation of xenobiotic defense and biotransformation enzyme genes in *Oikopleura *will have to be clarified further in future studies. Based on comparison with mechanisms in vertebrates and other invertebrates, and BaP-modulated gene expression analysis and genome survey, we propose a schematic model for possible mechanisms involved (Figure 7). In vertebrate systems, induction of biotransformation enzymes in response to xenobiotic stress involves both xenobiotic receptor-mediated (AhR and nuclear receptors) and non-receptor mediated mitogen-activated protein kinase (MAPK) pathways [47,48]. No AhR was detected in *Oikopleura*, but it is possible that some of the expanded NR1I and NR1H related members of nuclear receptors serve as xenobiotic receptors and regulate the transcription of some of the biotransformation genes such as CYPs. In vertebrates, many anti-oxidant and phase II biotransformation enzyme genes are targets of the transcription factors such as Nrf2, activated by signaling mechanisms involving MAPK pathways [30,49,50]. Components of the Nrf2 signaling pathway appear to be conserved in *Oikopleura *(Additional file 1, Figure S2, Table 1). Indeed, Nrf2 signaling is one of the top pathways significantly affected by BaP (Table 3A), suggesting its involvement in the regulation of anti-oxidant protein genes including GSTα, GCLc and GCLm up-regulated by BaP (Additional file 3). Both Nrf2 and AP-1 factors are involved in oxidant induced transcriptional regulation of GCLc and GCLm genes [51]. The MAPK pathway is also involved in the regulation of the transcription factor HSF1 in response to cellular stress [52]. BaP induction of many genes in the component pathways indicated in Figure 7 suggests that this model likely applies also in *Oikopleura*. For example, the transcription factors HSF1/4 and JunD (component of AP-1 complex), CYP3-like-2 gene, antioxidant genes and ABC transporters were up-regulated here by BaP (Additional file 3).

## Conclusions

*Oikopleura *appears to have basic defensome toolkit or genes for biotransformation enzymes for responding to xenobiotic stressors, although it may lack some key genes playing roles in vertebrate responses. AhR, not detected in *Oikopleura*, may have no important toxicological role outside vertebrates. Xenobiotic induced biotransformation and anti-oxidant enzyme gene expression responses to BaP treatment in *Oikopleura *show similarities to those observed in vertebrates, with the important exception of an AhR signaling mediated responses. The pattern of responses to Clo treatment appears different between *Oikopleura *and vertebrates.

## Methods

### Identification and sequence analysis of *Oikopleura *genes

*Oikopleura *genes were identified by both Hidden Markov Model (HMM) searches (HMMER 3.0, http://hmmer.org/) in predicted *O. dioica *protein database in NCBI using respective PFAM models of conserved domains, and by BLAST searches in the *Oikopleura *genome (sequenced to 14X coverage) and EST (about 300,000) databases. *Mus musculus *Swiss-Prot CYPs retrieved from NCBI database were used in BLAST searches and *Oikopleura *CYP hits were then re-BLASTed to identify all conserved CYPs in the genome. Then we compared with CYPs identified by profile Hidden Markov Model (HMM) searches and identified CYP gene models were manually curated. PAS domain proteins were identified by a combination of HMM searches and BLAST searches using *Mus musculus, Danio reiro, Caenorhabditis elegans, Branchiostoma floridae, Ciona intestinalis, Strongylocentrotus purpuratus *and *Nematostella vectensis *proteins retrieved from the NCBI database.

HMM searches and BLAST searches (using mouse Swiss-Prot sequences retrieved from the NCBI database) in *O. dioica *genome and EST databases were also used to identify transcription factors involved in Nrf2 pathway and other putative oxidative stress response signaling transcription factors (Additional file 1, Figure S2). Protein sequences for other organisms were retrieved from the NCBI database.

Phylogenetic tree analyses were performed on the Phylogeny.fr platform http://www.phylogeny.fr[53]. Multiple alignments were performed using MUSCLE. Alignments were manually edited and PhyML and TreeDyn programs were used to construct and visualize phylogenetic trees, respectively.

### Chemical exposure

Preliminary experiments were performed to estimate concentration ranges and exposure times that induced gene expression changes (as determined using qPCR and RNA blot assays of few genes) without causing excess mortality using clofibrate (Clo) (Sigma-Aldrich, St. Louis, MO) and benzo[a]pyrene (BaP) (Sigma-Aldrich). In these experiments 0.2 μM BaP and 5 μM Clo were found to be optimal concentrations. The 24 hour LC50 value of Clo was found to be approximately 14 μM. BaP has very low solubility in seawater and we did not determine its LC50. About 130 four-days-old sexually immature animals (*O. dioica*) were placed in 1L glass beakers with filtered and UV-light treated seawater, fed with algal culture and kept in suspension by rotation of a paddle connected to an electric motor [8]. The compounds were dissolved in 1 ml Dimethyl sulfoxide (DMSO), diluted in 50 ml seawater, vigorously vortexed and added. DMSO alone (1 ml) was used as a control. Final concentrations of Clo in seawater were 1 μM (0.243 mg/L) and 5 μM (1215 mg/L). Because of very low solubility of BaP, the actual concentrations in the seawater were not known. BaP was added at 0.2 μM (50.4 μg/L) and 1 μM (252 μg/L) nominal concentrations. There were no large and visible precipitates in the 0.2 μM BaP concentration, although invisible precipitates should form. Precipitates formed in the 1 μM concentration of BaP that formed larger aggregates possibly due to enhanced nucleation. The aggregation appeared to have resulted in lower up-take of BaP by the animals in the 1 μM concentration than in the lower (0.2 μM) concentration. Thus, the animals in the higher BaP concentration were swimming normal and less stressed than animals exposed to the lower (0.2 μM) concentration. Well dispersed tiny precipitates in the 0.2 μM concentration appear to have been taken up by the animals which filter microscopic food particles and ingest. The animals were kept at room temperature and harvested after 10 hrs of exposure by picking using inverted plastic pipettes, rinsed in filtered seawater and centrifuged 3 min at 12,000 rpm. Then the pellet was frozen in liquid nitrogen and stored at -80°C until RNA extraction.

### RNA extraction and dscDNA synthesis

Total RNA was isolated from the frozen animals using the RNeasy Mini Kit according to manufacturer's protocols (QIAGEN, Hilden, Germany). RNA concentration and quality was assessed using NanoDrop ND-1000 (NanoDrop Technologies, Wilmington, DE), and agarose gel electrophoresis. For each sample, 5 μg total RNA was converted to dscDNA using SuperScript Double-Stranded cDNA Synthesis Kit (Invitrogen, Carlsbad, CA), and dscDNA samples were submitted for microarray analysis.

### Whole genome tiling arrays

Custom made *O. dioica *whole genome tiling arrays manufactured by Roche NimbleGen (NimbleGen, Madison, WI) for the Sars International Centre for Marine Molecular Biology were used for gene expression analysis using a competitive hybridization strategy. The oligonucleotide features (represented by over 2 million probe sequences, with a probe size range between 50-75 nucleotides) cover the entire 70 Mb genome with average overlap between adjacent probes of approximately 30 bases. Thus, each gene in the genome was represented by numerous overlapping probes along its entire length. Labeling (using Cy3-coupled random nonamers for treated samples and Cy5-coupled random nonamers for DMSO control samples), and two-color hybridization (using 15 μg of each labeled sample per array) and scanning using an Axon 4000B scanner (Axon Instruments Inc., Union City, CA) were performed at the University of Iowa using standard conditions described in the Gene Expression User Guide (Roche NimbleGen).

### Tiling array data analysis

Array data generated from each group (including controls hybridized to the same array) were quantile normalized separately. Background subtraction was performed by subtracting the top 2% of random probe (negative control) intensity values from individual *O. dioica *probe intensity values. These random probes (which represent 13,936 of the 2,173,626 features on the array) were included on the array to help define background probe intensity levels. Only probes with positive intensity values after background subtraction were included in the analysis. For a gene to be considered as expressed, at least 50 percent of the probes covering exons of the gene had to have a positive intensity. A gene was assigned with a median intensity of the positive probes covering it. T-test was used to test whether there is any significant difference (alpha level = 0.05) between means of two groups (treated versus DMSO control). MIAME complaint array data has been deposited in NCBI's Gene Expression Omnibus (GEO) database (GEO accession GSE33818).

Genes with sum of the normalized median intensities (DMSO control and treated) lower than 3000 were further removed from the analysis because the signals were found to be unreliable after inspection of expression profiles using SignalMap browser (NimbleGen). To identify differentially regulated genes, the normalized median intensities were used to calculate expression ratios (treated/control) for each predicted gene. Genes with significantly different intensity levels (threshold p-value = 0.05, t-test) and with at least 1.5 fold expression changes were considered differentially regulated between treated and control groups. Applying the cut-off values, largely the 0.2 μM BaP and 5.0 μM Clo concentration samples were used for analysis of significant differential expression. The 1.0 μM BaP and 1.0 μM Clo were not optimal concentrations in inducing differential gene expressions. However, largely overlapping sets of differentially regulated genes were detected for the two concentrations of each compound with "dose-response" trends in the majority of cases. Less than 10% of the significantly differentially regulated genes were specifically regulated by 1.0 μM but not 0.2 μM BaP or 1.0 μM but not 5 μM Clo, and these were also included in the list of differentially regulated genes applying more stringent p-values (threshold p-value = 0.01, t-test). For genes for which the predicted protein was annotated with the best BLAST hit (see below), the analysis resulted in 762 (336 up-regulated and 426 down-regulated) and 630 (166 up-regulated and 464 down-regulated) genes differentially regulated by BaP and Clo, respectively. In addition, 425 and 446 genes that were not annotated (BLAST e-value ≥ 0.001) were differentially regulated by BaP and Clo, respectively.

### Annotation and functional classification of differentially regulated genes

Predicted protein sequences of 17,112 genes in *Oikopleura *Genome Browser V3 https://www.genoscope.cns.fr/secure-nda/Oikopleura/cgi-bin/gbrowse/Oikopleura/ were BLAST searched against the mouse proteome in NCBI database (NCBI BLASTP) and each predicted protein was named according to the best mouse hit (E-value < 0.001). Thus, all subsequent pathway and functional analyses were performed on the mouse homologs (the best mouse BLASTP hits) of the differentially regulated *O. dioica *genes using mouse annotation databases. This was done because most tools available for functional and pathway analyses are developed mainly for mammalian model organisms and do not allow direct inputs of *Oikopleura *genes. The differentially regulated genes were analyzed using Gene Ontology (GO), KEGG and other tools in DAVID Bioinformatics Resources [34,35]. The mouse protein_gi_accessions for 762 BaP-differentially regulated genes (Additional file 3) and 630 Clo-differentially regulated genes (Additional file 4) were submitted to DAVID database http://david.abcc.ncifcrf.gov/ and analyzed using mouse proteome background and default settings. In addition, the gene lists were subjected to more detailed pathway, process and network analyses using the MetaCore knowledge database and software suite (GeneGo, St. Joseph, MI) [36]. Predicted proteins with insufficient homologies to mouse proteins (BLASTP E-value ≥ 0.001) that accounted for 36% of BaP regulated genes (Additional file 9) and 41% of Clo regulated genes (Additional file 10) were not included in pathway analyses.

### Quantitative real-time PCR (qPCR)

qPCR was performed for validation of differential expressions in the microarray experiments. A total of 14 genes found to be differentially regulated using microarray by one or both compounds (0.2 μM BaP and 5 μM Clo) were analyzed. One of each primer pair (Additional file 1, Table S4) was designed to span exon-exon junctions to avoid amplification of genomic DNA. The amplicon sizes were 80-150 bp. RNA polymerase B11a (RPB11a) (Additional file 1, Table S4) was used as a house-keeping gene for normalization (reference gene). In preliminary experiments using the same RNA samples, comparison of RPB11a with beta actin showed both are good housekeeping reference genes. The same RNA samples analyzed by microarrays were used. For each sample, total RNA (1.0 μg) was reverse-transcribed using SuperScript III First-Strand Synthesis System for RT-PCR in 20 μL reaction as described in the manufacturer's protocols (Invitrogen). Then the reaction was diluted 1:10, and 5 μL of the cDNA was used in 20 μL amplification reaction using FastStart SYBR Green Master Mix according manufacturer's protocols (Roche, Basel, Switzerland), and qPCR was performed using LightCycler 480 Real-Time PCR System (Roche). The thermal cycling conditions were as follows: an initial denaturation of 95°C for 10 min, followed by 45 cycles of denaturation at 95°C for 10 s and an annealing temperature of 55°C for 20 s and elongation at 72°C for 30 s. Negative controls with no reverse transcriptase enzyme were run for each primer pair and each sample was amplified in duplicate. Post-amplification melting curve analysis was performed to check specificity of products. The PCR products were also analyzed by agarose gel electrophoresis to verify the amplification of a single product of the right size. The relative quantification method [54] was used to calculate gene expression and expression level for each gene was expressed as fold-change relative to the DMSO control.

## List of abbreviations

BaP: benzo[a]pyrene; Clo: Clofibrate; CYP: cytochrome P450; AhR: aryl hydrocarbon receptor; PAH: polycyclic aromatic hydrocarbon; NR: nuclear receptor; SXR: steroid and xenobiotic receptor; PXR: pregnane × receptor; CAR: constitutive androstane receptor; PPARα: peroxisome proliferator-activated receptor alpha; ARNT: AhR nuclear translocator; bHLH: basic-helix-loop-helix; PAS: Per-ARNT-Sim; Nrf2: nuclear factor (erythroid-derived 2)-like 2; HSF: heat shock factor; HIF: hypoxia-inducible factor; Clock: circadian locomoter output cycles protein kaput; VDR: vitamin D receptor; LXR: liver-X-receptor; FXR: farnesoid-X-receptor; Keap1: kelch-like ECH-associated protein 1; cnc-bZIP: cap 'n' collar-basic leucine-zipper; ARE: anti-oxidant response element; HSP: heat-shock protein; GCL: glutamate-cysteine ligase; TCA: tricarboxylic acid cycle; ROS: reactive oxygen species; GST: glutathione-s-transferase; ABC: ATP-binding cassette; MAPK: mitogen-activated protein kinase; MKK7: MAPK kinase7; PKC-A: protein kinase C-A; AP-1: activator protein 1; GCLm: GCL modifier subunit; GCLc: GCL catalytic subunit; GGT: gamma-glutamyltransferase; TXN: thioredoxin; HMM: Hidden Markov Model; DMSO: Dimethyl sulfoxide.

## Authors' contributions

FY designed and carried out the experiments, sequence and microarray data analyses and drafted the manuscript. SB carried out the microarray hybridization experiments. HEF and JMB participated in the exposure and qPCR experiments. OAK participated in genome survey and sequence analysis. FD performed transcriptome sequence mappings and analyses. RM carried out statistical analysis of tiling array data. CC, JRM and EMT performed tiling array development and analysis. AG and DC participated in the conception, design and coordination of the study, participated in sequence analysis and helped to draft the manuscript. All authors read and approved the final manuscript.

## Supplementary Material

Additional file 1 **Supplemental figures and tables**. Additional_file1.doc contains Figures S1-S7 and Tables S1-S4. Click here for file

Additional file 2 **Sequences of *O. dioica *CYP proteins**. Additional_file2.txt is a list of sequences and accessions for *O. dioica *CYPs. *O. dioica *(Od) CYPs are tentatively named after putative vertebrate homologs at family levels and numbered consecutively. Click here for file

Additional file 3 **A list of differentially regulated genes by BaP used in pathway analyses**. Additional_file3.xls contains 762 genes differentially regulated by BaP (736 genes differentially regulated by 0.2 μM BaP, at least 1.5 folds with t-test threshold p-value = 0.05, and 26 genes differentially regulated only by 1 μM BaP, at least 1.5 folds with threshold p-value = 0.01). For each predicted *O. dioica *protein, the name and accession number of the best BLAST hit in *M. musculus *is shown. "Ratio" indicates ratios of expression levels (BaP treated mean intensity/DMSO control mean intensity). Differentially regulated genes with BLASTP E-value ≥ 0.001 are not included here and listed separately in Additional file 9. Click here for file

Additional file 4 **A list of differentially regulated genes by Clo used in pathway analyses**. Additional_file4.xls contains 630 genes differentially regulated by Clo (607 genes differentially regulated by 5 μM Clo, at least 1.5 folds with t-test threshold p-value = 0.05, and 23 genes differentially regulated only by 1 μM Clo, at least 1.5 folds with threshold p-value = 0.01). For each predicted *O. dioica *protein, the name and accession number of the best BLAST hit *M. musculus *protein is shown. "Ratio" indicates expression levels (Clo treated mean intensity/DMSO control mean intensity). Differentially regulated genes with BLASTP E-value > 0.001 are not included here and listed separately in Additional file 10. Click here for file

Additional file 5 **Figure legend**. Additional_file5.pdf contains detailed figure legend for MetaCore (GeneGo) pathway maps and networks http://www.genego.com/. Click here for file

Additional file 6 **Selected networks built from BaP-regulated genes using *Transcription Regulation *algorithm in MetaCore**. Additional_file6.xls contains a list networks built from BaP-regulated genes using *Transcription Regulation *algorithm. The list of the mouse homologs of genes differentially regulated by BaP (Additional file 3) was uploaded as the input list for generation of biological networks in MetaCore (GeneGo) using *Transcription Regulation *algorithm with default settings. This is a variant of the shortest paths algorithm with main parameters of 1. relative enrichment with the uploaded data, and 2. relative saturation of networks with canonical pathways.. In this workflow the networks are prioritized based on the number of fragments of canonical pathways on the network. Click here for file

Additional file 7 **Selected over-connected networks built from BaP-regulated genes using *Analyze *algorithm in MetaCore. **Additional_file7.xls contains a list of over-connected networks built from BaP-regulated genes. The full list of the mouse homologs of genes differentially regulated by BaP (Additional file 3) was uploaded for generation of biological networks in MetaCore using *Analyze *network algorithm with default settings. This is a variant of the shortest paths algorithm with main parameters of 1. relative enrichment with the uploaded data, and 2. relative saturation of networks with canonical pathways. In this workflow the networks are prioritized based on the number of fragments of canonical pathways on the network. Click here for file

Additional file 8 **Selected over-connected networks built from Clo-regulated genes using *Analyze *algorithm in MetaCore. **Additional_file8.xls contains networks built from Clo-regulated genes. The full list of the mouse homologs of genes differentially regulated by Clo (Additional file 3) was uploaded for generation of biological networks in MetaCore using *Analyze *network algorithm with default settings. Click here for file

Additional file 9 **A list of genes differentially regulated by BaP with insufficient similarities to mouse sequences**. Additional_file9.xls contains 425 genes differentially regulated by BaP with insufficient similarities to mouse proteome (BLASTP E-value ≥ 0.001) and not used in pathway analyses. Click here for file

Additional file 10 **A list of genes differentially regulated by Clo with insufficient similarities to mouse sequences**. Additional_file10.xls contains 446 genes differentially regulated by Clo with insufficient similarities to mouse proteome (BLASTP E-value ≥ 0.001) and not used in pathway analyses. Click here for file
