# Supplementary material for: Conservation and divergence of chemical defense system in the tunicate Oikopleura dioica revealed by genome wide response to two xenobiotics
Source: BMC Genomics. 2012 Feb 2;13:55. doi: 10.1186/1471-2164-13-55 (PMC3292500; doi:10.1186/1471-2164-13-55)
Supplement: Additional file 1 — Supplemental figures and tables. Additional_file1.doc contains Figures S1-S7 and Tables S1-S4. [file 1471-2164-13-55-S1.DOC]

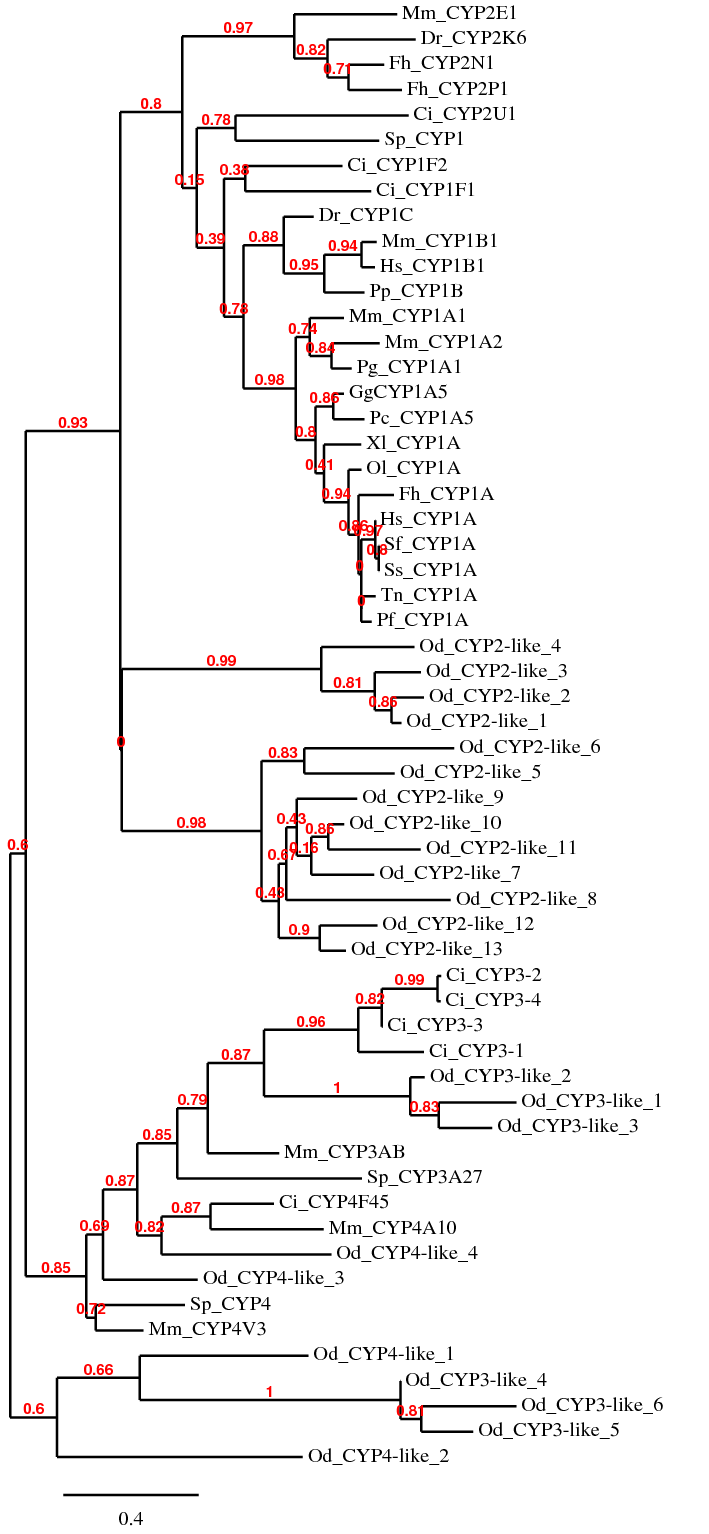


## Figure S1 - Maximum likelihood phylogenetic tree for Oikopleura CYPs

*O. dioica* (Od) CYPs are tentatively named according to families they appear to be most similar to and numbered consecutively. See Additional file 2 for the full list of *O. dioca* CYP protein sequences and their accessions. Sequences from other organism (mainly CYP1 families) are taken from . Other sequences were retrieved from NCBI database.

Branch support values are shown at the nodes.

Abbreviations: Od, O*. dioica;*  Nv, *N. vectensis*; Sp, *S. purpuratus*; Ci, *C. intestinalis*; Dr, ; Fh, *Fundulus heteroclitus;* Hs,*Homo sapiens*; Pp, *Pleuronectes platessa*; Pc, *Phalacrocorax carbo*; Gg, *Gallus gallus*; Sf, *Salvelinus fontinalis*; Ss, *Salmo salar*; Tn, *Tetraodon nigroviridis*; Pf, *Platichthys flesus*; Pg, *Phoca groenlandica*;Xl, *Xenopus laevis;* Ol, *Oryzias latipes*.

**
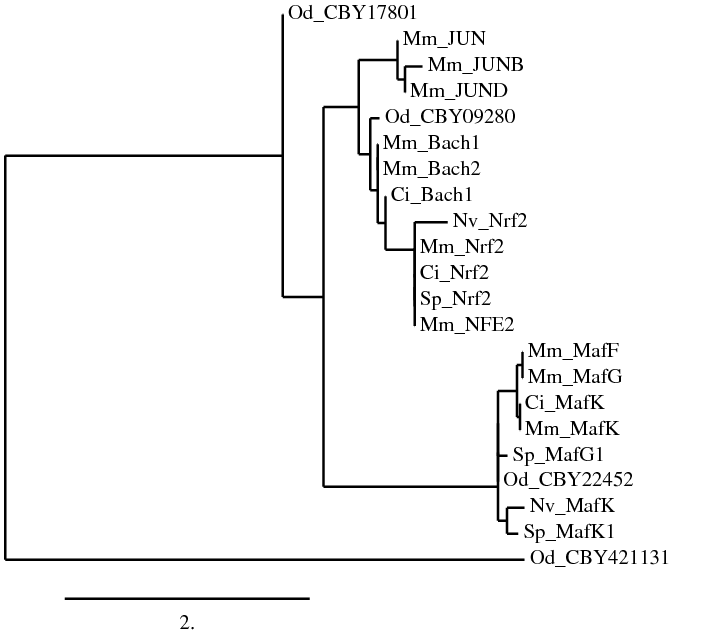
**

## Figure S2 - Maximum likelihood phylogenetic tree for O. dioca candidate small maf proteins, Nrf2, Bach and JUN proteins.

Abbreviations: Od, O*. dioica;* Mm, *Mus musculus*; Nv, *N. vectensis*; Sp, *S. purpuratus*; Ci, *C. intestinalis*.


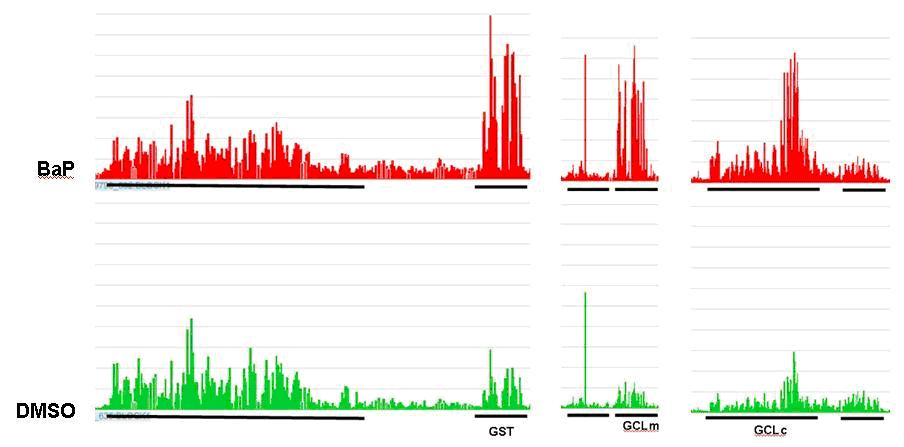


## Figure S3 - Tiling array expression profiles

Expression levels of glutathione S-transferase alpha (GST), glutamate cysteine ligase modifier subunit (GCLm) and glutamate cysteine ligase catalytic subunit (GCLc) in DMSO control (in green) and 0.2 μM BaP treated (in red) *O. dioica.* Underlines indicate gene models on scaffolds. In each case, an unaffected neighbouring gene on the scaffold is shown as a house-keeping control.


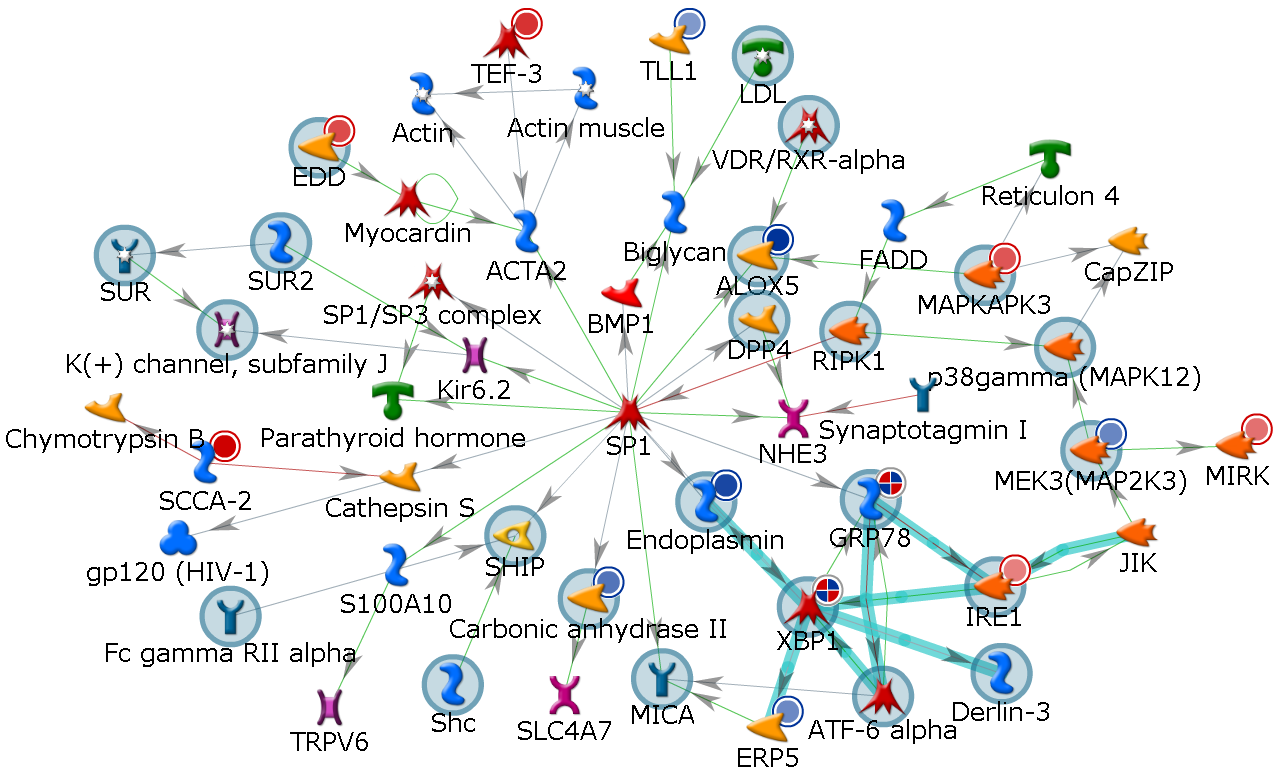


## Figure S4 - The third ranking network generated from BaP modulated gene list.

Biological networks were built using Analyze algorithm in MetaCore (GeneGo) with default settings. Up‑regulated genes are marked with red circles; down‑regulated with blue circles. The 'checkerboard' color indicates mixed expression for the gene between multiple tags for the same gene. Genes involved in GO Biological Process “stress response” are marked by bigger light blue circles. Thick cyan lines indicate part of GO Biological Process “endoplasmic reticulum unfolded protein response”. See Additional file 5 for detailed figure legend.


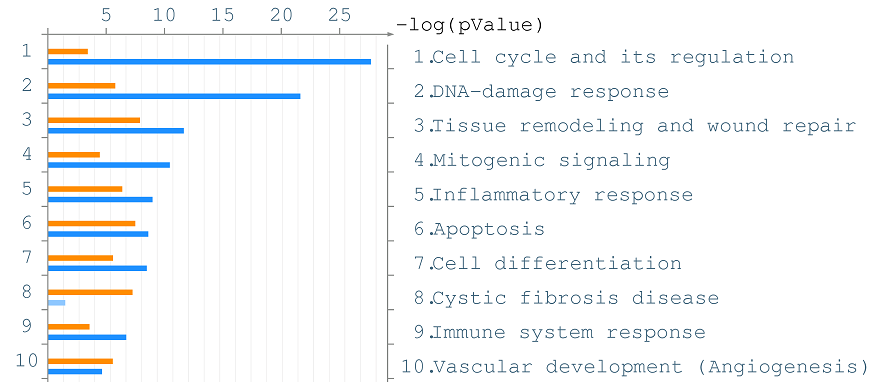


**A**


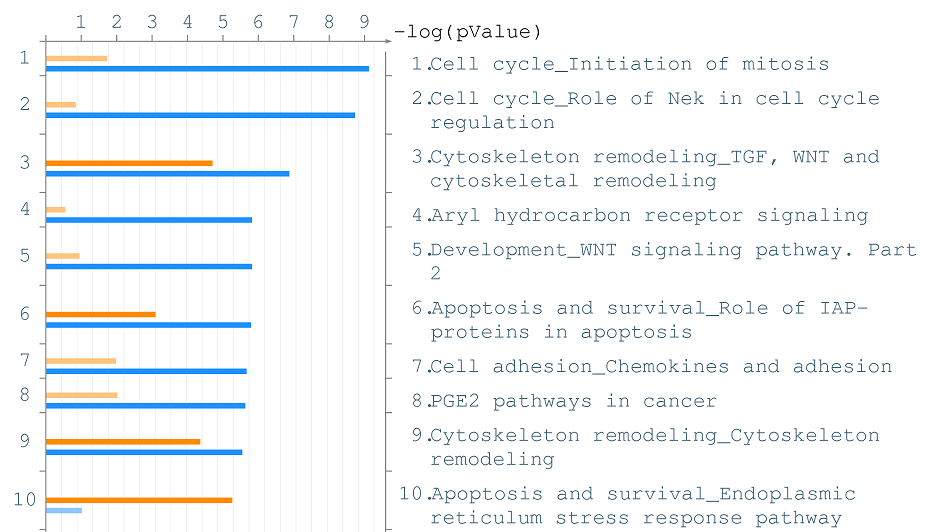


**B**

## Figure S5 - GeneGo Map Folders (A) and Pathway maps (B) for genes differentially regulated by BaP in Oikopleura (orange bars) and human cells (blue bars).

Only the top 10 significant (FDR = 0.05) map folders are shown. Light orange and light blue bars indicate values that are not statistically significant. The dotted rectangle in blue in the pathway maps (B) highlights Aryl hydrocarbon receptor (AhR) signaling which is among significantly enriched pathways in human cells but not in *Oikopleura*. Analysis was performed in MetaCore (GeneGo). Human cells microarray data was from .

**
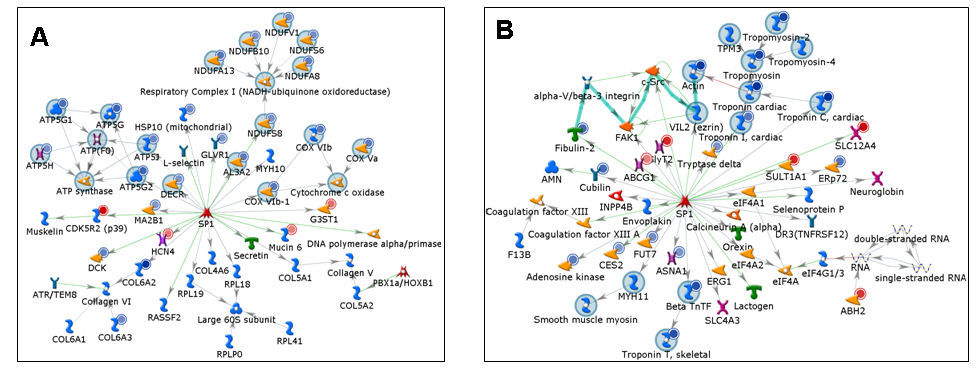
**

## Figure S6 - Networks generated from Clo modulated gene list.

Biological networks were built using Analyze algorithm in MetaCore (GeneGo) with default settings. Up‑regulated genes are marked with red circles; down‑regulated with blue circles. (**A**) First ranking network, genes involved in GO Biological Process “oxidative phosphorylation” are marked by bigger light blue circles. (**B**) Third ranking network, genes involved in GO Biological Process “muscle contraction” are marked by bigger light blue circles. Thick cyan lines indicate fragments of canonical pathways. See Additional file 5 for detailed figure legend.


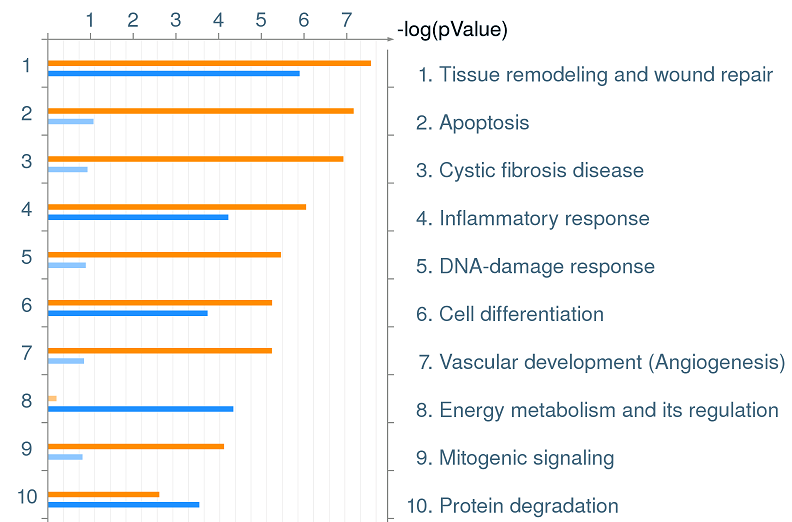


**A**


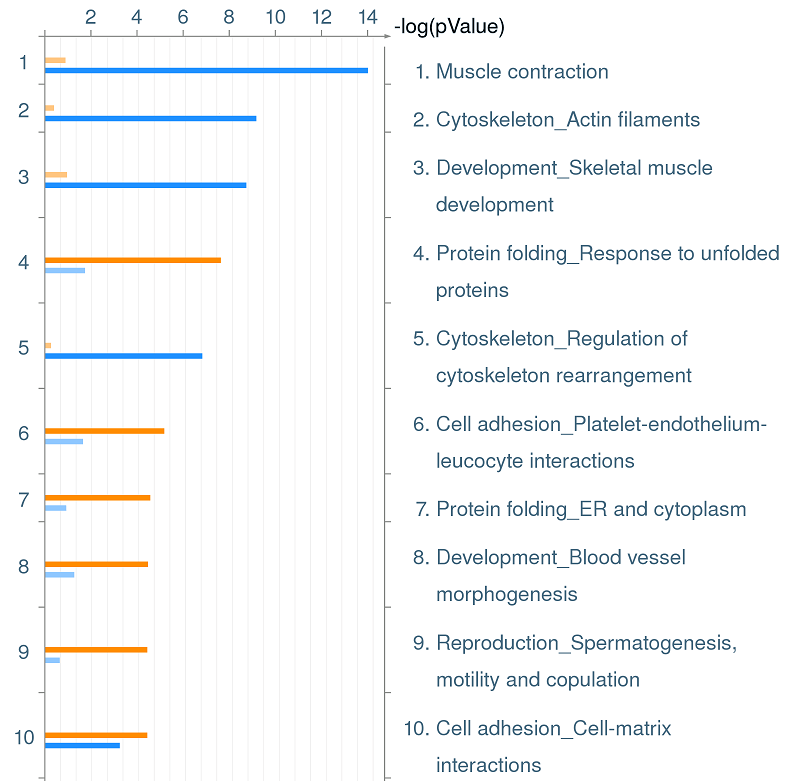


**B**


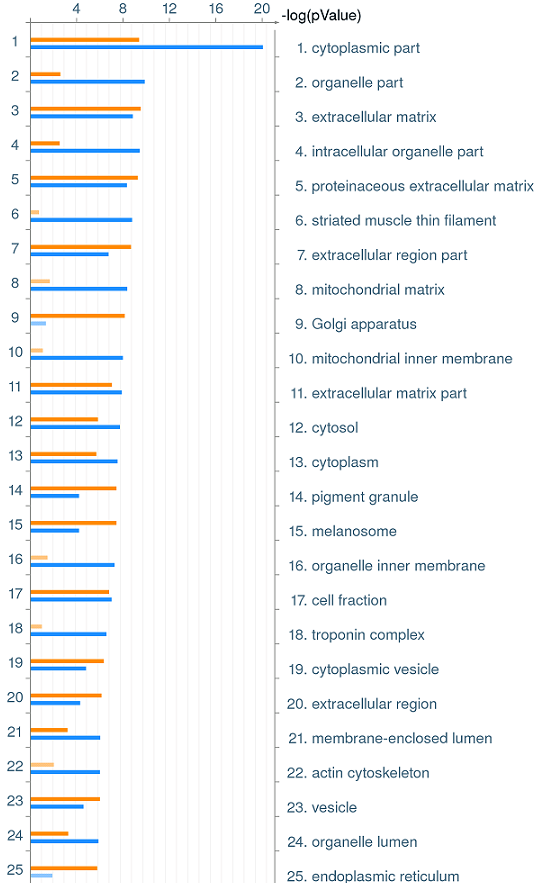


**C**

## Figure S7 - Comparison of BaP (orange bars) and Clo (blue bars) top Map Folders (A) Process Networks (B) and GO_Localizations (C).

Deep orange and deep blue bars indicate statistically significant values at (FDR = 0.05). Light orange (8 in **A**; 1, 2, 3, 5 in **B**; 6, 8, 10, 16, 18, 22 in **C**) and light blue bars indicate values that are not statistically significant. Analyses were performed in MetaCore (GeneGo).

## Table S1 - BaP up-regulated heat shock protein (HSP) genes

| ***O. dioica* accession** | **Accession** | **Description** | **Raio (0.2µ M BaP)** |
| --- | --- | --- | --- |
| CBY12775 | XP_002157141 | small hsp, partial [*Hydra magnipapillata*] | 21.4 |
| CBY12776 | XP_002160804 | similar to small hsp [*H. magnipapillata*] | 14.5 |
| CBY12817 | XP_002130038 | hsp 67B2 [*C. intestinalis*] | 10.3 |
| CBY24006 | CAG12424 | HSP70 [*T. nigroviridis*] | 9.5 |
| CBY10802 | NP_001157997 | rhodanese-like domain containing 1 (RHOD_HSP67B2) | 6.2 |
| CBY15022 | BAE38016 | HSP70 protein [*M. musculus*] | 4.6 |
| CBY19557 | XP_002124705 | similar to hypoxia up-regulated 1 (HSP70 protein) [*C. intestinalis*] | 2.3 |
| CBY20002 | NP_034607 | 60 kDa HSP, mitochondrial [*M. musculus*] | 2.0 |
| CBY17856 | BAE27553 | HSP 90-alpha [*M. musculus*] | 1.9 |
| CBY17857 | BAE40342 | HSP 70 kDa [*M. musculus*] | 1.4 |
| CBY21997 | NP_001029696 | stress-70 protein, mitochondrial precursor [*Bos taurus*] | 1.7 |

## Table S2 - Oikosins down regulated by BaP and Clo treatments

| **Accession** | **Gene name** | **Ratio (0.2µM BaP)** | **Raio (5µM Clo)** |
| --- | --- | --- | --- |
| CAC83956 | Oikosin 4C protein [*O. dioica*] | 0.16 | 0.50 |
| CAC83955 | Oikosin4B [*O. dioica*] | 0.30 | 0.38 |
| CAC83957 | Oikosin 5A protein [*O. dioica*] | 0.34 | 0.53 |
| CAC40992 | Oikosin2A protein [*O. dioica*] | 0.40 | 0.52 |
| CAC83956 | Oikosin 4C protein [*O. dioica*] | 0.11 | 0.33 |
| CAC83954 | Oikosin 4A protein [*O. dioica*] | 0.35 | 0.46 |

# Table S3A - Genes down-regulated by both BaP and Clo

| **Putative name** | ***O. dioica* accession** | **Ratio (BaP)** | | **Ratio (Clo)** | |
| --- | --- | --- | --- | --- | --- |
|  |  | **0.2 μM** | **1 μM** | **1 μM** | **5 μM** |
| caveolin-3 | CBY20090 | 0.64 | 0.68 | 0.57 | 0.49 |
| EF-hand domain-containing protein 1 | CBY20539 | 0.59 | 0.71 | 0.79 | 0.63 |
| RIKEN cDNA E330026B02 gene | CBY20796 | 0.36 | 0.34 | 0.86 | 0.51 |
| mCG12867, isoform CRA_c | CBY21001 | 0.36 | 0.33 | 0.82 | 0.54 |
| tectorin beta | CBY16364 | 0.54 | 0.56 | 0.77 | 0.41 |
| heparan sulfate 2-O-sulfotransferase 1 | CBY12357 | 0.34 | 0.57 | 1.06 | 0.50 |
| troponin C, slow skeletal and cardiac muscles | CBY12556 | 0.62 | 0.67 | 0.76 | 0.22 |
| creatine kinase S-type, mitochondrial precursor | CBY18300 | 0.65 | 0.78 | 0.79 | 0.60 |
| meprin 1 beta | CBY07476 | 0.20 | 0.54 | 0.78 | 0.36 |
| deleted in malignant brain tumors 1 | CBY07479 | 0.36 | 0.62 | 0.74 | 0.47 |
| peroxidasin homolog (Drosophila) | CBY07512 | 0.44 | 0.64 | 0.74 | 0.52 |
| CAP-GLY domain containing linker protein 2 | CBY07696 | 0.47 | 0.46 | 0.90 | 0.63 |
| arylsulfatase I precursor | CBY12752 | 0.46 | 0.68 | 0.69 | 0.48 |
| solute carrier family 11, member 2 | CBY12894 | 0.58 | 0.61 | 1.01 | 0.54 |
| PGAPDH isoform 1 | CBY12928 | 0.37 | 0.48 | 0.84 | 0.57 |
| inter-alpha trypsin inhibitor, heavy chain 1 | CBY07761 | 0.28 | 0.46 | 0.65 | 0.34 |
| eosinophil peroxidase | CBY07839 | 0.51 | 0.76 | 0.76 | 0.52 |
| peroxidasin homolog (Drosophila) | CBY18616 | 0.52 | 0.80 | 0.83 | 0.41 |
| cubilin precursor | CBY13017 | 0.33 | 0.53 | 0.93 | 0.39 |
| meprin A subunit beta precursor | CBY08119 | 0.28 | 0.63 | 0.79 | 0.22 |
| ER-Golgi intermediate compartment protein 1 | CBY08126 | 0.64 | 0.62 | 0.97 | 0.49 |
| tubulin, alpha 1C | CBY08200 | 0.47 | 0.51 | 0.98 | 0.50 |
| mCG140834 | CBY08231 | 0.50 | 0.62 | 0.85 | 0.57 |
| galactose-4-epimerase, UDP | CBY08252 | 0.54 | 0.72 | 0.90 | 0.63 |
| sorbitol dehydrogenase | CBY08272 | 0.53 | 0.67 | 0.76 | 0.31 |
| phospholipase A2 precursor | CBY18931 | 0.39 | 0.66 | 0.84 | 0.47 |
| solute carrier family 15, member 2 | CBY18965 | 0.64 | 0.83 | 0.71 | 0.42 |
| membrane metallo endopeptidase | CBY18972 | 0.52 | 0.64 | 0.78 | 0.48 |
| Fc fragment of IgG binding protein | CBY13343 | 0.19 | 0.36 | 0.55 | 0.12 |
| epidermal growth factor | CBY13475 | 0.43 | 0.71 | 0.95 | 0.32 |
| tectorin alpha | CBY13524 | 0.21 | 0.44 | 0.67 | 0.21 |
| tectorin alpha | CBY08296 | 0.39 | 0.68 | 0.92 | 0.40 |
| maltase-glucoamylase | CBY13650 | 0.66 | 0.66 | 0.73 | 0.46 |
| enhancing factor | CBY13670 | 0.61 | 0.57 | 0.68 | 0.56 |
| kallikrein B, plasma 1 | CBY13717 | 0.57 | 0.72 | 0.95 | 0.46 |
| protease, serine, 7 (enterokinase) | CBY08820 | 0.39 | 0.64 | 0.81 | 0.49 |
| heparan sulfate 2-O-sulfotransferase 1 | CBY06711 | 0.24 | 0.52 | 0.91 | 0.38 |
| unnamed protein product | CBY06736 | 0.36 | 0.45 | 0.76 | 0.64 |
| uridine phosphorylase 1 | CBY06747 | 0.36 | 0.52 | 0.75 | 0.63 |
| protein disulfide isomerase associated 4 | CBY06760 | 0.46 | 0.45 | 0.63 | 0.41 |
| collagen, type VI, alpha 1 | CBY06810 | 0.53 | 0.68 | 0.74 | 0.35 |
| fibrillin 1 | CBY06834 | 0.48 | 0.67 | 0.86 | 0.51 |
| mCG121902 | CBY06879 | 0.51 | 0.76 | 0.68 | 0.66 |
| pyruvate carboxylase | CBY06961 | 0.42 | 0.54 | 0.71 | 0.45 |
| laminin subunit gamma-1 precursor | CBY07094 | 0.99 | 1.43 | 0.76 | 0.53 |
| tenascin-X | CBY07142 | 0.29 | 0.65 | 0.80 | 0.51 |
| RIKEN cDNA 1110001D15 gene | CBY07224 | 0.41 | 0.57 | 0.54 | 0.24 |
| predicted gene 1008 | CBY07226 | 0.33 | 0.55 | 0.73 | 0.36 |
| procollagen C-endopeptidase enhancer 2 | CBY07307 | 0.43 | 0.62 | 0.64 | 0.33 |
| aldehyde dehydrogenase 16 family, member A1 | CBY13933 | 0.62 | 0.72 | 1.12 | 0.47 |
| collagen, type VI, alpha 2 | CBY13979 | 0.23 | 0.45 | 0.65 | 0.13 |
| meprin A subunit beta precursor | CBY14075 | 0.44 | 0.61 | 0.97 | 0.66 |
| glycoprotein 2 (zymogen granule membrane) | CBY14430 | 0.53 | 0.70 | 0.99 | 0.30 |
| cubilin precursor | CBY14604 | 0.36 | 0.51 | 0.85 | 0.34 |
| selectin, endothelial cell | CBY14616 | 0.45 | 0.66 | 1.05 | 0.63 |
| EGF-like module containing, mucin-like sequence 1 | CBY09278 | 0.64 | 0.51 | 0.76 | 0.21 |
| IGF binding protein, acid labile subunit | CBY24167 | 0.43 | 0.73 | 0.84 | 0.46 |
| similar to fibrillin 2; fibrillin 2 | CBY19233 | 0.61 | 0.64 | 0.81 | 0.45 |
| La ribonucleoprotein domain family, member 6 | CBY09493 | 0.26 | 0.44 | 0.90 | 0.43 |
| inter-alpha trypsin inhibitor, heavy chain 3 | CBY09533 | 0.19 | 0.42 | 0.75 | 0.36 |
| similar to Protein C21orf63 homolog precursor | CBY09547 | 0.26 | 0.83 | 0.74 | 0.27 |
| similar to Protein C21orf63 homolog precursor | CBY09551 | 0.29 | 0.75 | 0.77 | 0.32 |
| transmembrane protease, serine 8 (intestinal) | CBY19480 | 0.47 | 0.63 | 0.72 | 0.32 |
| unnamed protein product | CBY19515 | 0.65 | 0.73 | 0.81 | 0.44 |
| tissue specific transplantation antigen P35B | CBY21096 | 0.54 | 0.82 | 1.07 | 0.60 |
| deleted in malignant brain tumors 1 | CBY21183 | 0.08 | 0.35 | 0.58 | 0.31 |
| collagen alpha-4(VI) chain precursor | CBY21316 | 0.93 | 1.05 | 0.65 | 0.62 |
| protein disulfide isomerase associated 3 | CBY21606 | 0.63 | 0.47 | 0.77 | 0.51 |
| phospholipase A2, group IIA | CBY14625 | 0.17 | 0.34 |  | 0.13 |
| unnamed protein product | CBY14648 | 0.44 | 0.61 | 0.70 | 0.42 |
| transmembrane serine protease 8 precursor | CBY17757 | 0.58 | 0.67 | 1.02 | 0.47 |
| cholinergic receptor, nicotinic, alpha polypeptide 10 | CBY14764 | 0.41 | 0.51 | 0.94 | 0.25 |
| meprin A subunit beta precursor | CBY14773 | 0.44 | 0.60 | 0.94 | 0.54 |
| bicaudal C homolog 1 (Drosophila) | CBY15039 | 0.45 | 0.75 | 1.00 | 0.52 |
| enteropeptidase isoform 1 | CBY24357 | 0.61 | 0.79 | 0.78 | 0.46 |
| peptidylprolyl isomerase B | CBY09658 | 0.30 | 0.32 | 0.40 | 0.34 |
| predicted gene 4848 | CBY09673 | 0.47 | 0.69 | 0.71 | 0.36 |
| prospero-related homeobox 1 | CBY09674 | 0.48 | 0.58 | 0.74 | 0.55 |
| unnamed protein product | CBY09721 | 0.60 | 0.50 | 0.72 | 0.37 |
| ephrin B3 | CBY10042 | 0.49 | 0.60 | 0.84 | 0.48 |
| enhancing factor | CBY10157 | 0.54 | 0.54 | 0.87 | 0.44 |
| unnamed protein product | CBY21744 | 0.29 | 0.58 | 0.75 | 0.67 |
| butyrylcholinesterase | CBY21799 | 0.40 | 0.51 | 0.93 | 0.58 |
| collagen, type XXIV, alpha 1 | CBY22354 | 0.24 | 0.29 | 0.69 | 0.44 |
| Fc fragment of IgG binding protein | CBY15138 | 0.14 | 0.33 | 0.48 | 0.11 |
| fibulin 2 | CBY15209 | 0.39 | 0.62 | 1.01 | 0.36 |
| cubilin precursor | CBY15356 | 0.41 | 0.52 | 0.87 | 0.36 |
| transmembrane protease serine 9 | CBY10663 | 0.61 | 0.79 | 0.91 | 0.51 |
| Fc fragment of IgG binding protein | CBY10684 | 0.26 | 0.45 | 0.62 | 0.24 |
| polycystic kidney disease protein 1-like 2 precursor | CBY24766 | 0.28 | 0.62 | 0.81 | 0.29 |
| fibrocystin-L precursor | CBY24943 | 0.94 | 1.85 | 0.71 | 0.30 |
| dihydropyrimidinase | CBY24949 | 0.50 | 0.61 | 0.89 | 0.52 |
| endoplasmin | CBY22718 | 0.43 | 0.39 | 0.63 | 0.38 |
| transmembrane protease, serine 9 | CBY15554 | 0.22 | 0.48 | 0.94 | 0.52 |
| unnamed protein product | CBY15799 | 0.41 | 0.65 | 0.92 | 0.45 |
| ovochymase 2 | CBY10743 | 0.31 | 0.66 | 0.79 | 0.35 |
| serine racemase | CBY10770 | 0.67 | 0.63 | 0.57 | 0.35 |
| meprin A subunit beta precursor | CBY10777 | 0.56 | 0.82 | 0.79 | 0.50 |
| Phactr2 protein | CBY10827 | 0.60 | 0.68 | 0.64 | 0.54 |
| Fc fragment of IgG binding protein | CBY10832 | 0.20 | 0.44 | 0.56 | 0.17 |
| CUB domain-containing protein 2 precursor | CBY10852 | 0.57 | 0.56 | 0.95 | 0.60 |
| group IID secretory phospholipase A2 precursor | CBY19707 | 0.47 | 0.54 | 0.76 | 0.45 |
| dehydrogenase/reductase (SDR family) member 10 | CBY19761 | 0.36 | 0.40 | 0.98 | 0.55 |
| dehydrogenase/reductase (SDR family) member 10 | CBY19763 | 0.30 | 0.42 | 1.00 | 0.55 |
| mCG13774, isoform CRA_b | CBY11054 | 0.53 | 0.50 | 0.71 | 0.37 |
| otoconin-90 precursor | CBY11075 | 0.56 | 0.63 | 0.82 | 0.48 |
| otoconin-90 precursor | CBY11077 | 0.56 | 0.71 | 0.58 | 0.39 |
| unnamed protein product | CBY11097 | 0.54 | 0.82 | 0.88 | 0.49 |
| branched chain ketoacid dehydrogenase E1 | CBY23283 | 0.63 | 0.79 | 0.73 | 0.59 |
| pro-epidermal growth factor precursor | CBY11219 | 0.56 | 0.58 | 0.82 | 0.54 |
| CNDP dipeptidase 2 (metallopeptidase M20 family) | CBY19824 | 0.51 | 0.60 | 0.81 | 0.51 |
| alpha-(1,3)-fucosyltransferase isoform b | CBY11287 | 0.46 | 0.64 | 0.81 | 0.51 |
| transmembrane protease, serine 5 (spinesin) | CBY23385 | 0.28 | 0.49 | 0.77 | 0.39 |
| meprin 1 beta | CBY23446 | 0.22 | 0.60 | 0.87 | 0.37 |
| peroxidasin homolog (Drosophila) | CBY23580 | 0.24 | 0.72 | 0.83 | 0.34 |
| fucosyltransferase 4 | CBY11529 | 0.56 | 0.53 | 1.04 | 0.50 |
| arylsulfatase i | CBY11544 | 0.35 | 0.49 | 0.97 | 0.45 |
| double von Willebrand factor A domains | CBY11552 | 0.51 | 0.61 | 0.58 | 0.22 |
| galactose-3-O-sulfotransferase 3 | CBY11728 | 0.36 | 0.57 | 1.02 | 0.46 |
| collagen, type VI, alpha 3 | CBY11793 | 0.47 | 0.38 | 0.89 | 0.50 |
| phospholipase A2, group IID | CBY11829 | 0.25 | 0.52 | 0.83 | 0.42 |
| mCG126042 | CBY18020 | 0.26 | 0.48 | 0.89 | 0.48 |
| enhancing factor | CBY12043 | 0.21 | 0.43 | 0.77 | 0.21 |
| astacin-like metalloendopeptidase (M12 family) | CBY12133 | 0.33 | 0.55 | 0.81 | 0.24 |
| plasminogen | CBY12175 | 0.34 | 0.54 | 0.60 | 0.22 |
| hypoxia up-regulated 1 | CBY19557 | 2.3 | 0.7 | 0.8 | 0.5 |

# Table S3B - Genes up-regulated by both BaP and Clo

| **Putative name** | ***O. dioica* accession** | **Ratio (BaP)** | | **Ratio (Clo)** | |
| --- | --- | --- | --- | --- | --- |
|  |  | **0.2 μM** | **1 μM** | **1 μM** | **5 μM** |
| telomerase associated protein 1 | CBY20166 | 2.30 | 1.87 | 1.33 | 1.65 |
| deleted in malignant brain tumors 1 | CBY20493 | 1.51 | 1.04 | 1.15 | 1.72 |
| IQ motif containing GTPase activating protein 2 | CBY16294 | 7.34 | 3.80 | 1.64 | 1.37 |
| trans-acting transcription factor 3 | CBY18499 | 3.66 | 1.20 | 1.12 | 1.51 |
| homogentisate 1, 2-dioxygenase | CBY18563 | 1.65 | 1.38 | 1.78 | 2.25 |
| kin of IRRE like | CBY12779 | 2.28 | 1.46 | 1.67 | 1.14 |
| CD109 antigen | CBY07776 | 1.65 | 1.34 | 1.18 | 1.86 |
| cholinergic receptor, nicotinic, beta polypeptide 3 | CBY08370 | 6.95 | 3.19 | 1.17 | 1.57 |
| plectin 1 | CBY13941 | 6.89 | 2.33 | 1.42 | 1.63 |
| alkB, alkylation repair homolog 2 (E. coli) | CBY14094 | 3.94 | 3.03 | 1.65 | 1.42 |
| predicted gene 766 | CBY09415 | 1.62 | 1.61 | 1.51 | 1.50 |
| MAP kinase-activated protein kinase 3 | CBY21068 | 1.83 | 1.20 | 1.07 | 1.67 |
| mannose receptor, C type 2 | CBY21590 | 2.44 | 1.96 | 5.53 | 17.98 |
| glutathione S-transferase, alpha 1 (Ya) | CBY09928 | 2.63 | 1.50 | 1.70 | 2.52 |
| similar to syntenin; syndecan binding protein | CBY21990 | 1.72 | 1.56 | 1.16 | 2.05 |
| eukaryotic translation initiation factor 2C, 2 | CBY22129 | 2.29 | 1.00 | 1.12 | 1.60 |
| collagen, type XII, alpha 1 | CBY22190 | 3.25 | 1.66 | 1.14 | 1.78 |
| plasminogen activator inhibitor 1 precursor | CBY15357 | 2.14 | 1.59 | 1.50 | 1.57 |
| leukocyte receptor cluster (LRC) member 9 | CBY24678 | 5.26 | 2.30 | 1.67 | 1.77 |
| unnamed protein product | CBY15749 | 1.56 | 0.92 | 1.75 | 1.38 |
| major vault protein | CBY25003 | 3.94 | 3.14 | 1.55 | 2.24 |
| mannose receptor, C type 2 | CBY12121 | 3.17 | 1.83 | 4.93 | 16.13 |
| zonadhesin | CBY25135 | 1.87 | 1.85 | 1.54 | 1.42 |
| mCG134590, isoform CRA_b | CBY25012 | 0.6 | 2.4 | 1.2 | 2.1 |

# Table S3C - Genes oppositely regulated by BaP and Clo

| **Putative name** | ***O. dioica* accession** | **Ratio (BaP)** | | **Ratio (Clo)** | |
| --- | --- | --- | --- | --- | --- |
|  |  | **0.2 μM** | **1 μM** | **1 μM** | **5 μM** |
| unnamed protein product | CBY23918 | 0.6 | 0.7 | 1.1 | 1.7 |
| unnamed protein product | CBY08516 | 2.1 | 1.2 | 0.7 | 0.4 |
| laminin subunit gamma-1 precursor | CBY07094 | 1.0 | 1.4 | 0.8 | 0.5 |
| solute carrier family 7, member 9 | CBY13821 | 2.0 | 0.9 | 0.8 | 0.5 |
| myosin light chain kinase, smooth muscle | CBY13942 | 1.2 | 1.9 | 1.0 | 0.6 |
| glutamate dehydrogenase 1 | CBY24069 | 1.0 | 1.1 | 0.9 | 0.4 |
| laminin B1 subunit 1 | CBY14851 | 1.5 | 2.2 | 1.1 | 0.6 |
| laminin subunit alpha-5 precursor | CBY10267 | 1.3 | 1.7 | 0.9 | 0.6 |
| Golgi-associated plant pathogenesis-related protein 1 | CBY10667 | 1.2 | 2.1 | 0.7 | 0.3 |
| mCG141862, isoform CRA_c | CBY15801 | 0.6 | 0.7 | 1.5 | 1.1 |
| integrator complex subunit 6 | CBY15808 | 1.7 | 1.6 | 1.0 | 0.5 |
| carbonic anhydrase 4 | CBY12062 | 0.4 | 0.4 | 1.9 | 1.0 |

**Table S4 -** Primers used in qPCR

| **Putative name** | **Symbol** | ***O. dioica***  **accession** | **Forward (5’ to 3’)** | **Reverse (5’ to 3’)** |
| --- | --- | --- | --- | --- |
| G protein-coupled receptor 176 | GP176 | CBY20947 | CTTCAATTGCGCTTCTTGATCATT | AAAAGCGGGTGAAAGACCAGTC |
| Neuronal acetylcholine receptor subunit beta-3 | ACHB3 | CBY08370 | CATGGTCCCTGTAAAAAGCTCC | TCGAGCAATTTTGCCAGTCAAATG |
| SDR family member 10 | DHRS4 | CBY13552 | CTGTGAACGTCAAGGCGCCTAT | GCGTAACTCATCAATTTGTTAGTTC |
| glycine transporter type-2 | SC6A5 | CBY19189 | TTCGACAACAACATTGTCCGAGA | TGCGCCATGAAGCCGAGATAA |
| RIKEN cDNA 4931408A02 | CU063 | CBY09551 | TATCACCAAGTTCACAAAGGTGCT | ACGATTGTGATTTCCTTGGCTTGC |
| C-type mannose receptor 2 | MRC2 | CBY21590 | ATTGGACATACGGCTTTCGAGG | GTGGTTTCCACTGTCTCGACCAA |
| unnamed protein | ABCAD | CBY21184 | TCAGCACCATGTCGGACGTGT | AGCGAGCTTGTTCATAGCCTTTG |
| protein DDI1 homolog 2 | DDI2 | CBY22176 | GGATCTACGGGAACTACAGTAAA | TTCGATCTCCATGTCTTCACTCT |
| CUB/zona pellucida-like domains | PA2GD | CBY35250 | GGAACAGACGGAGACAGAGTT | CATCCCTGAATTTCGGAGTGG |
| cytochrome P450 3A13 | CP3AD | CBY21750 | TCGCTAATATGCAAGCGCGTC | GACCGATAAGGAATGTTGTAAG |
| Cytosolic 5'-nucleotidase 1B | 5NT1A | CBY22917 | GAAGCTCATTTTATGGCTGGCG | CGATTTGGTGTTGTCGACCTGG |
| gp250 precursor | SORL | CBY22510 | GCGAGCTCCTTTACACGGCTG | AAACTTTCGTTTGGCCCGGTG |
| Fc frag. of IgG binding protein | TECTA | CBY10832 | CGGTTCATTTTATCTTCTGAGGGA | CACCAGGGCCATCGAACAACAT |
| cytochrome P450, family 2, R1 | CP2R1 | CBY23488 | TTTCGGATACACTCCCGCATA | AACTGCAGCAAGAGATCCGAA |
| RNA polymerase II subunit (reference) | RPB11 | CBY18197 | AGGTTCTTTTCGCCGGCTAC | CTCTTCTAGCAGTGACAATTCAG |

References

1. Goldstone JV, Goldstone HMH, Morrison AM, Tarrant A, Kern SE, Woodin BR, Stegeman JJ: **Cytochrome p450 1 genes in early deuterostomes (tunicates and sea urchins) and vertebrates (chicken and frog): Origin and diversification of the CYP1 gene family.** *Molecular Biology and Evolution* 2007, **24:**2619-2631.

2. Sparfel L, Pinel-Marie M-L, Boize M, Koscielny S, Desmots S, Pery A, Fardel O: **Transcriptional Signature of Human Macrophages Exposed to the Environmental Contaminant Benzo(a)pyrene.** *Toxicological Sciences* 2010, **114:**247-259.
