# Supplementary figures and images for: Conservation and divergence of chemical defense system in the tunicate Oikopleura dioica revealed by genome wide response to two xenobiotics
Source: BMC Genomics. 2012 Feb 2;13:55. doi: 10.1186/1471-2164-13-55 (PMC3292500; doi:10.1186/1471-2164-13-55)

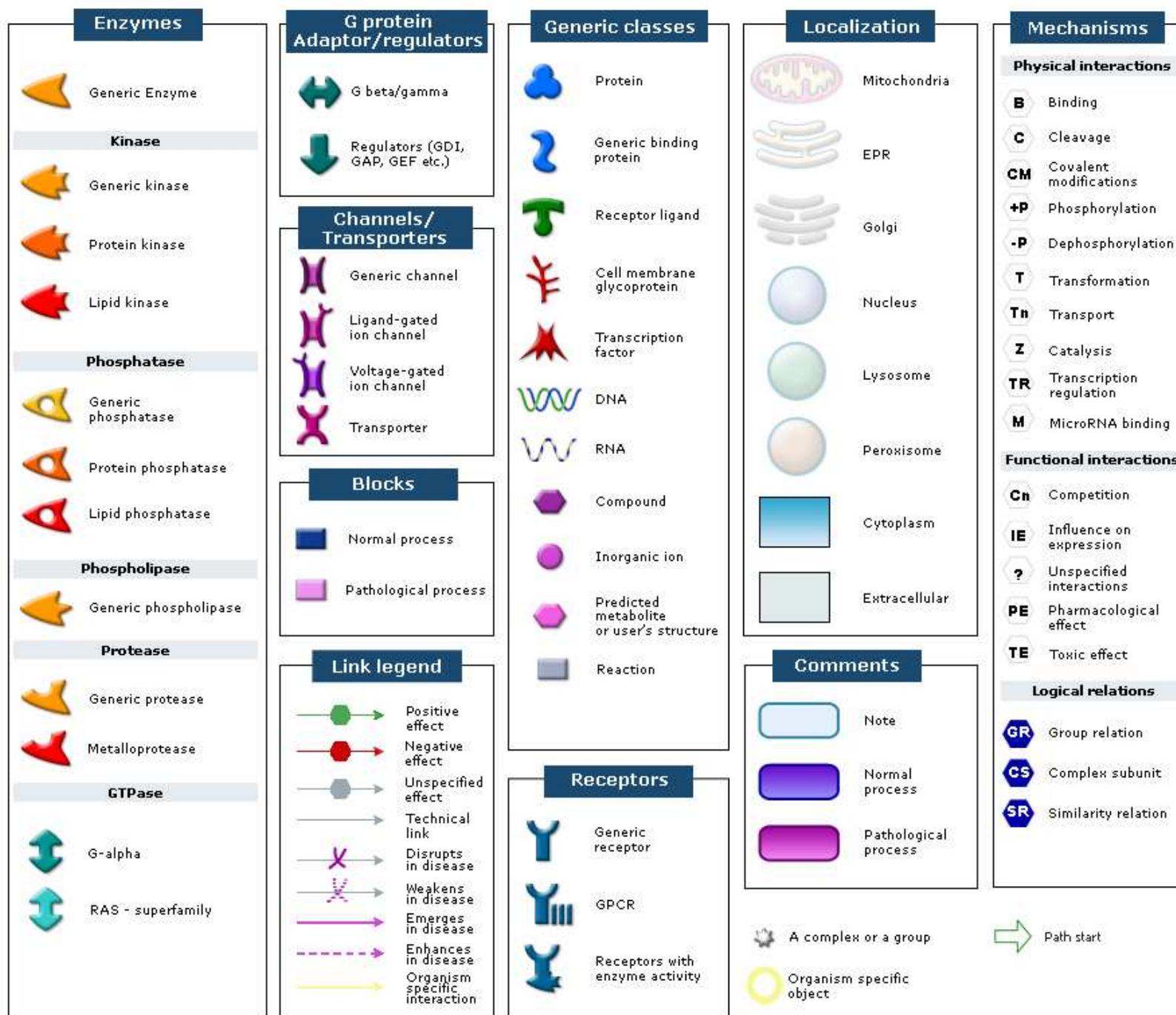

Supplement: Additional file 5 — Figure legend. Additional_file5.pdf contains detailed figure legend for MetaCore (GeneGo) pathway maps and networks http://www.genego.com/. [file 1471-2164-13-55-S5.PDF]
